# A dual-reporter LDLR system integrating fluorescence and luminescence for understanding LDLR regulation and facilitating drug discovery

**DOI:** 10.3389/fmolb.2025.1552085

**Published:** 2025-03-13

**Authors:** Peng Jiao, Na Yang, Qianfeng Jia, Baozhen Fan, Ke Feng, Jian Yu, Shengtian Zhao

**Affiliations:** ^1^ Department of Urology, Binzhou Medical University Hospital, Binzhou, Shandong, China; ^2^ Medical Integration and Practice Center, Shandong University, Jinan, Shandong, China; ^3^ Shandong Engineering Research Center of Molecular Medicine for Renal Diseases, Yantai, Shandong, China; ^4^ Department of Physiology, Binzhou Medical University, Yantai, Shandong, China; ^5^ Department of Urology, Yantai Affiliated Hospital of Binzhou Medical University, Yantai, Shandong, China; ^6^ Department of Basic Medical Education, Yantai Nursing School, Yantai, Shandong, China; ^7^ Shandong Provincial Engineering Laboratory of Urologic Tissue Reconstruction, Jinan, Shandong, China; ^8^ Department of Urology, Qilu Hospital, Cheeloo College of Medicine, Shandong University, Jinan, Shandong, China

**Keywords:** LDLR, knock-in cell line, CRISPR/Cas9, EGFP, gaussia luciferase

## Abstract

**Introduction:**

The low-density lipoprotein receptor (LDLR) is integral to cholesterol metabolism and cardiovascular health. Enhancing LDLR expression is a promising strategy for treating hyperlipidemia and reducing the risk of atherosclerosis. However, current LDLR reporter systems have limitations in detecting both transcriptional and translational regulation. To address this, we developed a novel dual-reporter LDLR system incorporating Enhanced Green Fluorescent Protein (EGFP) and Gaussia luciferase (Gluc) to enable precise monitoring of LDLR expression and function.

**Methods:**

A CRISPR/Cas9-mediated knock-in strategy was used to integrate EGFP and Gluc upstream of the stop codon located in exon 18 of the LDLR gene in HEK293 cells. The dual-reporter system allows real-time visualization of LDLR expression via EGFP fluorescence and quantitative assessment through secreted Gluc activity. The system was validated using western blotting, immunofluorescence, and functional assays, including DiI-LDL uptake and drug response analyses with statins and PCSK9 inhibitors.

**Results:**

The established LDLR-EGFP-Gluc knock-in cell line faithfully recapitulates endogenous LDLR expression and function. EGFP fluorescence accurately reflects LDLR expression dynamics, while Gluc activity provides a highly sensitive and quantitative readout. Functional assays confirmed that LDLR expression responds appropriately to statins and PCSK9 inhibitors. Additionally, screening for transcriptional regulators identified FOXP3 and CREB as novel modulators of LDLR expression, with CREB-mediated regulation involving the sterol regulatory element-binding protein 2 (SREBP2) pathway.

**Discussion:**

This dual-reporter system enables complementary monitoring of LDLR dynamics, providing enhanced sensitivity, accuracy, and versatility for studying LDLR regulation and function, as well as facilitating drug discovery targeting hyperlipidemia and cardiovascular diseases.

## 1 Introduction

The low-density lipoprotein (LDL) receptor (LDLR) is a membrane glycoprotein with a molecular mass of 160 kDa, that plays a crucial role in lipid metabolism and overall cardiovascular health (Li et al., 2001). LDLR regulates plasma cholesterol levels by removing LDL particles from the bloodstream, reducing cholesterol accumulation, and lowering the risk of atherosclerosis and cardiovascular diseases (Goldstein and Brown, 1985; Brown and Goldstein, 1986). Mutations or deficiencies in the LDLR gene are associated with genetic disorders like familial hypercholesterolemia (FH), which is characterized by high level of cholesterol and increased risk of early cardiovascular disease (Goldstein and Brown, 1973; Brown and Goldstein, 1974). Recent studies have demonstrated that up to 1.6% of patients at very high risk for cardiovascular disease are affected by familial hypercholesterolemia (FH), highlighting a direct association between LDLR and LDL cholesterol in the pathogenesis of cardiovascular disease (Dyrbuś et al., 2021). Both homozygous and heterozygous forms of familial hypercholesterolemia are marked by elevated levels of atherogenic LDL cholesterol, which contributes to the early onset of atherosclerotic cardiovascular disease (Ference et al., 2017).

LDLR is crucial for developing and optimizing cholesterol-lowering drugs and therapeutic strategies, making it a key focus in managing lipid balance and cardiovascular health (Nordestgaard et al., 2013; Srivastava, 2023). The agents currently available on the market for lowering LDL levels in individuals with hypercholesterolemia include statins and ezetimibe, among others (LaRosa et al., 1999; Yamamoto et al., 2006; Kastelein et al., 2008). Therefore, enhancing LDLR expression represents a potent treatment strategy for hypercholesterolemia. Consequently, there is a need for efficient and sensitive reporter assays to elucidate LDLR regulation and function under various conditions, screen for compounds that affect its activity, and model related diseases, all of which are essential for developing treatments for hyperlipidemia and cardiovascular diseases (Roger et al., 2012).

Despite the extensive research into LDLR’s role in lipid metabolism, the complexity of LDLR regulation is still not fully understood. LDLR expression is modulated through a combination of transcriptional regulation by the sterol regulatory element-binding protein 2 (SREBP-2) (Brown and Goldstein, 1997; Sun et al., 2007), post-translational regulation via PCSK9-mediated degradation (Qian Y. W. et al., 2007; Kwon et al., 2008), and post-transcriptional regulation involving the stability of LDLR mRNA (Wilson et al., 1998; Li et al., 2009). Current methods used to investigate the effects of lipid metabolism on LDL levels, as well as LDLR expression and activity, include the measurement of LDL uptake and degradation using 125I or fluorescence-labeled LDL, Western blotting, immunofluorescence microscopy, and flow cytometry (Romano et al., 2011; Etxebarria et al., 2012). Additionally, existing research tools, such as the LDLR promoter-luciferase reporter system, provide only a limited view of these complex regulatory mechanisms, mainly focusing on promoter activity in response to diverse conditions or drugs (Jasiecki et al., 2023).

To address these limitations, a new reporter cell line is needed that could adequately and faithfully mark LDLR with enhanced detection sensitivity and specificity which reflect both transcriptional and translational regulation of LDLR. Here, we introduce the Enhanced Green Fluorescent Protein (EGFP) and Gaussia luciferase (Gluc) genes upstream of the stop codon in exon 18, ensuring faithfully mimics endogenous LDLR expression and maintain all the original sequence of LDLR gene. Additionally, the EGFP and Gluc genes are fused to LDLR without the use of an internal ribosome entry site (IRES) or self-cleaving peptides. This approach was chosen because IRES-based systems can lead to differential expression between the reporter and the gene of interest, and P2A self-cleaving peptides can leave both a C-terminal extension on the upstream gene product and an N-terminal proline on the downstream product. By ensuring that all components are co-expressed as a single fusion protein, these constructs minimize the risk of such artifacts.

Gluc naturally contains an N-terminal signal peptide that directs its secretion through the classical endoplasmic reticulum (ER)-Golgi pathway. This signal peptide is responsible for the efficient secretion of Gluc into the extracellular medium, even when fused to other proteins (Thompson et al., 1989; Luft et al., 2014; Liaci et al., 2021). In our dual-reporter system, Gluc is fused to the C-terminal of LDLR-EGFP. Although Gluc is positioned at the C-terminal end, the presence of its native signal peptide allows it to be cleaved and secreted independently of the LDLR-EGFP fusion protein. In addition to the N-terminal signal peptide, Gluc can also be secreted through internal signal peptide-mediated mechanisms. Studies have shown that Gluc can be cleaved at internal signal peptide sites, leading to the release of the luciferase domain into the extracellular medium (Luft et al., 2014). This mechanism is independent of the N-terminal signal peptide and can occur even when Gluc is fused to other proteins.

This dual-reporter system provides fluorescence and luminescence readouts, respectively, enabling simultaneous and complementary monitoring of LDLR expression and cross-verification of results (Tannous, 2009). Gluc provides high sensitivity and a wide dynamic range for detecting low LDLR expression and offers precise, quantitative measurement useful for high-throughput screening and kinetic studies (Tannous et al., 2005; Wurdinger et al., 2008). EGFP fluorescence enables real-time visualization of LDLR expression, localization, and interactions within living cells, facilitating dynamic studies of receptor behavior. The combination of EGFP and Gluc allows for both immediate (EGFP) and longer-term (Gluc) monitoring of LDLR dynamics, which is useful for understanding how LDLR expression changes over time or in response to treatments. Using an LDLR reporter cell line with both EGFP and Gluc combines the strengths of fluorescence and luminescence measurements, providing a powerful tool for studying LDLR expression, regulation, and function in various experimental contexts. And this reporter cell line enhances sensitivity, accuracy, and versatility, making it a valuable asset for both basic research and applied studies in LDLR-targeting drug discovery and disease modeling.

## 2 Materials and methods

### 2.1 Cell lines

HEK293 and HepG2 cells were obtained from ATCC (United States) and cultured according to the distributor’s recommendations. The cells were cultured in Dulbecco’s modified Eagle’s medium (Gibco, United States) supplemented with 10% fetal bovine serum (Gibco), 1% penicillin-streptomycin and 1% L-glutamate (Hycyte, China). The cells were cultured in a humidified chamber with 5% CO_2_ at 37°C (Thermo Fisher, United States).

### 2.2 Plasmids

The Cas9 expression plasmid pX330, the overexpression plasmid pcDNA3.1, and the knockdown plasmid pLKO.1 were all purchased from OriGene Technologies (United States).

### 2.3 Construction of CRISPR/Cas9 plasmid

To construct the Cas9/sgRNA system, three sgRNAs were designed targeting the region upstream of the stop codon of the human LDLR gene using Integrated DNA Technologies (https://www.idtdna.com/). The sequences of the three sgRNA target sites are listed in Table 1. DNA oligonucleotides for each sgRNA were synthesized by Tsingke Biotechnology Co.,Ltd. (Tsingke Biotech, China).

**TABLE 1:** The primers used for this study.

Primer name	Sequence
LDLR-sgRNA1-F	CACCGGTCAGTCTGGAGGATGACG
LDLR-sgRNA1-R	AAACCGTCATCCTCCAGACTGACC
LDLR-sgRNA2-F	CACCGCAGAGACAGATGGTCAGTC
LDLR-sgRNA2-R	AAACGACTGACCATCTGTCTCTGC
LDLR-sgRNA3-F	CACCGAGACAGATGGTCAGTCTGG
LDLR-sgRNA3-R	AAACCCAGACTGACCATCTGTCTC
LDLR-genome-F	CTGGAGCAAACAGAGAGAGGG
LDLR-genome-R	CCTGTTCTGCCTCCCAGATG
LDLR-F	GAATCTACTGGTCTGACCTGTCC
LDLR-R	GGTCCAGTAGATGTTGCTGTGG
GAPDH-F	GTCTCCTCTGACTTCAACAGCG
GAPDH-R	ACCACCCTGTTGCTGTAGCCAA
pcDNA3.1-F	GAATTCTGCAGATATCCAGCAC
pcDNA3.1-R	GGATCCGAGCTCGGTACCAA
PCSK9-F	TTGGTACCGAGCTCGGATCCATGGGCACCGTCAGCTCCAG
PCSK9-R	GTGCTGGATATCTGCAGAATTCTCACTGGAGCTCCTGGGAGGC
YY1-F	TTGGTACCGAGCTCGGATCCATGGCCTCGGGCGACACCCT
YY1-R	GTGCTGGATATCTGCAGAATTCTCACTGGTTGTTTTTGGCCTTAGCATG
SREBF2-F	TTGGTACCGAGCTCGGATCCATGGACGACAGCGGCGAGCT
SREBF2-R	GTGCTGGATATCTGCAGAATTCTCAGGAGGCGGCAATGGCAGTG
AP-2a-F	TTGGTACCGAGCTCGGATCCATGAAAATGCTTTGGAAATTGACG
AP-2a-R	GTGCTGGATATCTGCAGAATTCTCACTTTCTGTGCTTCTCCTC
FOXP3-F	TTGGTACCGAGCTCGGATCCATGCCCAACCCCAGGCCTGG
FOXP3-R	GTGCTGGATATCTGCAGAATTCTCAGGGGCCAGGTGTAGGGT
STAT4-F	TTGGTACCGAGCTCGGATCCATGTCTCAGTGGAATCAAGTCC
STAT4-R	GTGCTGGATATCTGCAGAATTCTCATTCAGCAGAATAAGGAGACTTC
GR-F	TTGGTACCGAGCTCGGATCCATGGACTCCAAAGAATCATTAACTCC
GR-R	GTGCTGGATATCTGCAGAATTCTGAATAGCCATTAGAAAAAACTGTTCG
HOXD9-F	TTGGTACCGAGCTCGGATCCATGTTGGGTGGGAGTGCGGGA
HOXD9-R	GTGCTGGATATCTGCAGAATTCTCAGTCTCCTTTGGGGCATTTCTC
XBP1-F	TTGGTACCGAGCTCGGATCCATGGTGGTGGTGGCAGCCGC
XBP1-R	GTGCTGGATATCTGCAGAATTCTTAGTTCATTAATGGCTTCCAGCTTGGC
CREB-F	TTGGTACCGAGCTCGGATCCATGACCATGGAATCTGGAGC
CREB-R	GTGCTGGATATCTGCAGAATTCTTAATCTGATTTGTGGCAGTAAAGGTC
ELK1-F	TTGGTACCGAGCTCGGATCCATGGACCCATCTGTGACGCT
ELK1-R	GTGCTGGATATCTGCAGAATTCTCATGGCTTCTGGGGCCCTG
TBP-F	TTGGTACCGAGCTCGGATCCATGGATCAGAACAACAGCCT
TBP-R	GTGCTGGATATCTGCAGAATTCTTACGTCGTCTTCCTGAATCC
shRNA-FOXP3-F	CCGGCACACGCATGTTTGCCTTCTTCTCGAGAAGAAGGCAAACATGCGTGTGTTTTTG
shRNA-FOXP3-R	AATTCAAAAACACACGCATGTTTGCCTTCTTCTCGAGAAGAAGGCAAACATGCGTGTG
shRNA-CREB-F	CCGGACAGCACCCACTAGCACTATTCTCGAGAATAGTGCTAGTGGGTGCTGTTTTTTG
shRNA-CREB-R	AATTCAAAAAACAGCACCCACTAGCACTATTCTCGAGAATAGTGCTAGTGGGTGCTGT
shRNA-SREBF2-F	CCGGCCTCAGATCATCAAGACAGATCTCGAGATCTGTCTTGATGATCTGAGGTTTTTG
shRNA-SREBF2-R	AATTCAAAAACCTCAGATCATCAAGACAGATCTCGAGATCTGTCTTGATGATCTGAGG

The synthesized oligonucleotides were phosphorylate annealed by T4 PNK (NEB, United States). The mixture was heated to 95°C for 5 min and then gradually cooled to room temperature. The pX330 vector was digested with the BbsI (NEB, United States) restriction enzyme to create compatible overhangs for sgRNA insertion. Then the annealed sgRNA oligonucleotides were ligated into the BbsI-digested px330 vector using T4 DNA ligase (NEB). The successfully constructed pX330-sgRNA plasmids were named pX330-LDLR-sgRNAX (where X stands for 1–3). The sgRNA activity was assessed using the T7 endonuclease I (T7E1) assay.

### 2.4 Construction of donor plasmids

The donor plasmids contain a 285 bp left homologous arm, an EGFP sequence, a Gaussia luciferase sequence, a bGH poly(A) signal sequence, and a 285 bp right homologous arm, with EcoRI and BamHI (NEB) restriction enzyme sites at both ends of the sequence. The donor plasmids were synthesized by Tsingke Biotechnology Co.,Ltd. and cloned into the pUC57 vector. For cell transfection, the plasmids were linearized using EcoRI and BamHI double digestion and purified using the DNA Gel Extraction Kit (Tsingke Biotech).

### 2.5 T7E1 assay

Genomic DNA was extracted from HEK293 cells transfected with pX330-LDLR-sgRNAX and the corresponding pX330 control vector. This genomic DNA served as the template for amplifying the target sequence flanking the sgRNA binding sites. The target sequence was amplified by PCR (Thermo Fisher), and the resulting PCR products were purified using the DNA Gel Extraction Kit (Tsingke Biotech). The purified products were then denatured and reannealed to form heteroduplex DNA. These reannealed products were digested with T7EI endonuclease (Tsingke Biotech) and subsequently separated by DNA gel electrophoresis on a 1% agarose gel. The primers used for amplifying the target sequences in the T7E1 assay are listed in Table 1.

### 2.6 Cell transfection

To assess the cutting efficiency of the sgRNAs, HEK293 cells were seeded into 6-well plates 24 h prior to transfection. When the cell density reached 70%–80%, transfection was performed using Lipofectamine 3,000 reagent (Invitrogen, United States) according to the manufacturer’s instructions. A total of 2.5 μg of the pX330-LDLR-sgRNA plasmids or the corresponding control vector were transfected into the HEK293 cells. After 48 h, the cells were collected, and genomic DNA was extracted from both experimental and control groups using the Universal Genomic DNA Extraction Kit (Accurate Biotech, China). The extracted genomic DNA was subsequently used for the T7E1 assay.

### 2.7 Establishment of HEK293-LDLR-EGFP-Gluc-KI cell line

To establish a CRISPR/Cas9-mediated EGFP-Gluc knock-in reporter system under the control of the endogenous LDLR gene promoter in HEK293 cells (HEK293-LDLR-EGFP-Gluc knock-in cell line), 2 μg of pX330-LDLR-sgRNA plasmids and 2 μg of linearized donor plasmids were co-transfected into HEK293 cells in 6-well plates using Lipofectamine 3,000 transfection reagent (Invitrogen). After 48 h of transfection, EGFP-positive cells were sorted by flow cytometry (Beckman, United States).

### 2.8 Flow cytometry

After 48 h of transfection, HEK293 cells were collected and resuspended in PBS to generate a single-cell suspension. The cells were then sorted by flow cytometry to isolate EGFP-positive cells. These positive cells were sorted individually into 96-well plates, where single-cell clones were expanded. Genomic DNA was extracted from these clones, and PCR and sequencing were performed to verify the correct insertion of the donor plasmid into the reporter gene locus.

### 2.9 Quantitative real-time PCR (qRT-PCR)

Total RNA was extracted using Trizol reagent (Ambion, United States). According to the manufacturer’s instructions (Takara, Japan), total RNA was isolated and reverse transcribed using a reverse transcription system kit. cDNA quantification was performed using SYBR Green PCR Master Mix (ABI, United States) on the Thermo Fisher QuantStudio3 system. Gene expression levels were normalized to the expression of GAPDH. The sequences of the oligonucleotide primers are listed in Table 1.

### 2.10 Western blot

Total protein extracts were obtained by lysing cells on ice with RIPA lysis buffer (Sangon Biotech, China) containing protease and phosphatase inhibitors (Sangon Biotech). Protein concentrations were measured using the BCA Protein Assay (Takara). Under reducing conditions, protein expression was detected by SDS/PAGE. The membranes were blocked with 5% skim milk dissolved in TBST and incubated overnight at 4°C with primary antibodies, including LDLR Polyclonal Antibody (Proteintech, United States) and Gaussia Luciferase Polyclonal Antibody (Invitrogen, United States). Detection was achieved using HRP-conjugated secondary antibodies (Thermo Fisher), followed by ECL incubation (Thermo Fisher). All blot images were integrated using Adobe Illustrator 2021. Western blot quantification was performed using ImageJ.

### 2.11 Immunofluorescence staining

Cells were fixed on a confocal dish with 4% paraformaldehyde for 15 min and washed three times with PBS, each wash lasting 10 min. The cells were blocked with PBS containing 10% BSA at room temperature for 1 h. Cells were then incubated overnight at 4°C with LDLR Polyclonal Antibody (Proteintech), washed three times, and further incubated with Alexa FluorTM 647 secondary antibody (Invitrogen) for 1.5 h at room temperature. After three additional washes, nuclei were stained with DAPI (Sigma). Imaging was performed using a laser confocal microscope (Zeiss LSM 880, Germany). Immunofluorescence images were acquired and processed with Zen Software v2.3 (Blue Edition).

### 2.12 Construction of shRNA vectors

For each target gene to be knocked down, three pairs of 21-mer targets were designed using an siRNA selection tool. The corresponding oligonucleotides were synthesized as follows: Forward oligo: CCGG-21bp sense-CTCGAG-21bp antisense-TTTTTG and Reverse oligo: AATTCAAAAA-21bp sense-CTCGAG-21bp antisense. The synthesized oligos were annealed using a gradient annealing process. The pLKO.1 vector was digested with EcoRI and AgeI (NEB), followed by DNA gel extraction. The annealed oligos were then ligated into the gel-extracted pLKO.1 vector using T4 DNA ligase. Successfully constructed vectors were verified by sequencing and designated as shRNA plasmids. These three constructed vectors were transfected into cells, and the knockdown efficiency was assessed by qRT-PCR. The vector with the highest knockdown efficiency was used for subsequent experiments.

### 2.13 CCK-8 assay

Approximately 15,000 cells per well were seeded into 96-well culture plates. After incubation for 0, 12, 24, 48, and 72 h, 10 μL of CCK-8 reagent (Vazyme, China) was added to each well. The plates were then incubated at 37°C for 2 h, and absorbance was measured at 450 nm using a microplate reader.

### 2.14 DiI-LDL uptake assay

After 48 h of transfection, HepG2 cells were incubated with culture media containing 20 μg/mL DiI-LDL (Yiyuan Biotech, China) at 37°C for 4 h. Following incubation, the media containing DiI-LDL were removed, and the cells were washed several times with PBS. Fluorescence was measured using a microplate reader with excitation at 554 nm and emission at 571 nm. For immunofluorescence, cells were washed with PBS and fixed with 4% paraformaldehyde (PFA) for 15 min, followed by three washes with PBS. The cell nuclei were stained with DAPI for 5 min. After mounting, imaging was performed using a confocal microscope (Leica, Germany), and immunofluorescence intensity was quantified using ImageJ.

### 2.15 Cycloheximide assay

Cells were treated with Cycloheximide (CHX) (MCE, United States) at a final concentration of 50 μg/mL, and supernatants were collected at 0, 4, 8, and 12 h to measure luciferase activity. Simultaneously, cell lysates were collected, and the expression of LDLR was analyzed by Western blot. The protein levels were quantified to assess the effect of CHX on luciferase activity and LDLR protein expression.

### 2.16 Statistics

All the replicate experiments were performed at least three times. All results are expressed as mean values along with their respective standard error of mean. The statistical significance in the pairwise comparisons was evaluated by Student’s t-test and multiple groups by one-way ANOVA with Tukey’s *post hoc* test. p < 0.05 was denoted as a significant difference (*), p < 0.01 was classified as a highly significant difference (**) and p < 0.001 was classified as an extremely difference (***).

## 3 Results

### 3.1 Generation of the LDLR-EGFP-Gluc knock-in HEK293 cell line

**FIGURE 1:**
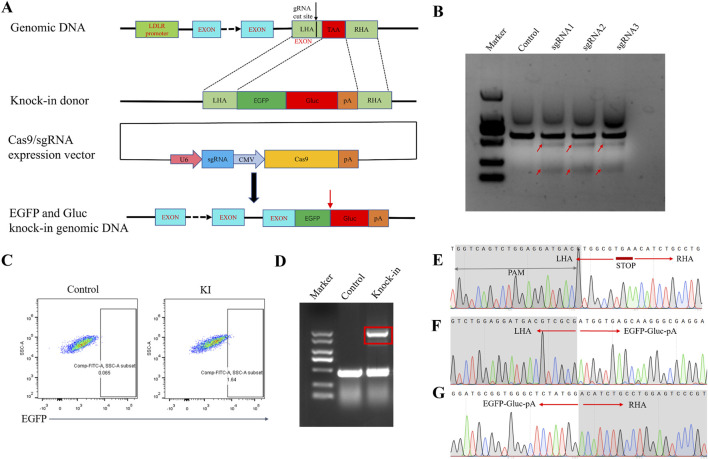
Generation of the HEK293 LDLR-EGFP-Gluc knock-in cell line. **(A)** Schematic diagram of the establishment of the EGFP-Gluc reporter gene cell line targeting the LDLR gene locus via homologous recombination. The sgRNA target site is located in the region between the last exon and the stop codon of the human LDLR gene. EGFP-Gluc is fused with LDLR through homologous recombination. The Cas9 and sgRNA expression vector is pX330. After integration, EGFP-Gluc is positioned before the stop codon of LDLR in the genome. Red arrows indicate the cleavage site between EGFP and Gluc. **(B)** T7E1 assay to detect the cutting efficiency of sgRNA. The cutting efficiencies of the pX330 control plasmid and the three pX330-LDLR-sgRNA plasmids. Red arrows indicate the expected positions of the DNA bands cleaved by T7E1. **(C)** Flow cytometry sorting of EGFP-positive cells. **(D)** PCR detection of donor insertion in sorted single-cell clones. Control represents wild-type HEK293 cells, and knock-in represents single-cell clones with successfully inserted donor plasmid. **(E)** Sequencing results near the LDLR stop codon in the genome of wild-type HEK293 cells **(F, G)** Sequencing results of the forward and reverse orientations in the HEK293 reporter gene cell line after insertion of the EGFP-Gluc sequence.

We employed a dual detector cassette comprising open reading frames EGFP and Gluc, integrated directly upstream of the STOP codon located in exon 18 of the LDLR gene that would be transcribed in tandem with LDLR and subject to the same regulation (Figure 1A). Three sgRNAs were selected. All sgRNAs were targeting the sequence near the stop codon in exon 18 to enable a C-terminal knock-in of the EGFP and GLuc coding sequence. The cutting efficiency of the sgRNAs was analyzed using the T7E1 assay, and the sgRNA with the highest efficiency, sgRNA1, was chosen for further experiments (Figure 1B). The donor vector contained the coding sequence of EGFP and Gluc flanked by sequences (∼300 bp) homologous to those directly upstream and downstream of the STOP codon of LDLR sequence. A new STOP codon for the fusion protein followed downstream of Gluc sequence.

Although the knock-in efficiency was low, indicated approximately 1.6% EGFP + cells in flow cytometry, the correctly edited cells could be enriched by fluorescence-activated cell sorting (FACS) (Figure 1C). Next, single cell clones were generated from the FACS-selected EGFP + population. To verify proper insertion of the reporter gene, total genomic DNA was extracted from the sorted reporter cells for junctional PCR analysis. Primers targeting the EGFP gene and endogenous sequences flanking the targeted insertion site confirmed the insertion of the reporter immediately downstream of the stop codon at exon 18. For most tested clones, we detected several PCR products corresponding to the size of the wild-type allele amplicon and knock-in allele amplicon, indicating a mono-allelic integration (Figure 1D). Sanger sequencing of all tested knock-in alleles revealed exact matches with the predicted sequences for a successful knock-in, and confirmed a precise excision of the positive selection cassette. The selected cell populations contained chimeric mRNA (target gene, fluorescence tag, and Gluc tag), indicating that a substantial number of cells of the population had undergone the intended integration events (Figures 1E–G).

### 3.2 Knock-in EGFP-Gluc does not alter cell morphology and function

**FIGURE 2:**
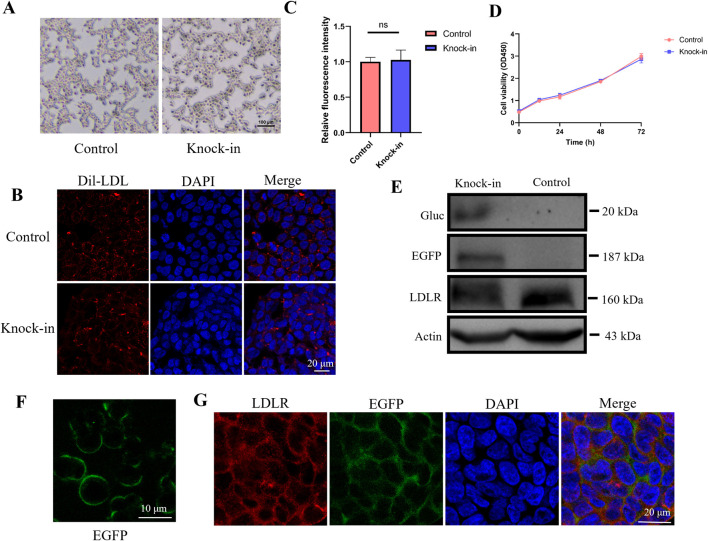
EGFP fluorescence intensity recapitulates endogenous LDLR. **(A)** Cell morphology of wild-type HEK293 and knock-in cell line. Scale bar, 100 μm. **(B, C)** LDL particle uptake was observed using confocal microscopy, and the fluorescence intensity in the cells was quantified (n = 4). **(D)** Cell viability assessment of wild-type HEK293 and knock-in cell line using the CCK8 assay. **(E)** Western blot analysis was performed to detect the expression of Gluc, LDLR, and LDLR-EGFP fusion protein in the knock-in cell line, using anti-Gluc and anti-LDLR antibodies. **(F)** Expression of EGFP in the knock-in cell line observed by confocal microscopy. Scale bar, 10 μm. **(G)** EGFP fluorescence and LDLR fluorescent staining were detected by immunofluorescence staining.

Microscopic observations revealed that both wild-type and knock-in HEK293 cells exhibited similar morphology, suggesting that the expression of EGFP and Gluc does not impact cell morphology (Figure 2A). Next, we examined whether the knock-in EGFP-Gluc affects cellular uptake function. After adding DiI-labeled low-density lipoprotein (Dil-LDL) to the culture medium, LDL particle uptake was observed using confocal microscopy, and the fluorescence intensity in the cells was quantified (Figures 2B, C). The results showed no significant difference in LDL uptake between the knock-in and control HEK293 cells, indicating that the knock-in of EGFP-Gluc does not affect the uptake function. Cell viability was assessed using the CCK-8 assay in both control and knock-in HEK293 cells. The results showed no significant difference in cell proliferation between the two groups, suggesting that the knock-in of EGFP-Gluc does not affect cell viability (Figure 2D).

### 3.3 EGFP fluorescence intensity recapitulates endogenous LDLR

It has been shown before that internal signal peptides have the potential to result in secretion of the downstream gene and can be cleaved by signal peptidase (Luft et al., 2014; Liaci et al., 2021). We enriched supernatants from serum-free media and performed immuno-blotting of cell lysates and enriched supernatants. A strong band at ∼20 kDa was observed in supernatants, that corresponds in size to wild-type Gluc, no band corresponding to full length LDLR-EGFP-Gluc was observed in the supernatant, indicating the protein needs to be cleaved for secretion (Figure 2E). To assess whether EGFP fluorescence accurately reflects endogenous LDLR expression dynamics in the knock-in cell line, we measured EGFP and LDLR protein expression levels using whole-cell lysates via Western blotting. We observed robust expression of both EGFP and LDLR in the reporter-transduced cells. In the cell lysates, LDLR (160 kDa) was detected in both the knock-in and wild-type HEK293 cells. Additionally, a band at 187 kDa was detected in the knock-in cell line, corresponding to the size of the LDLR-EGFP fusion protein, which is labeled as EGFP in Figure 2E. These results indicate that the LDLR-EGFP-Gluc fusion protein is expressed in the knock-in cell line, and since Gaussia luciferase is a secreted enzyme, its expression can be detected in the culture supernatant. Confocal microscopy showed that EGFP was correctly expressed on the cell membrane (Figure 2F). Next, we conducted immunofluorescence staining using anti-LDLR antibody to evaluate whether EGFP and LDLR are expressed simultaneously. As shown in Figure 2G, EGFP fluorescence and LDLR fluorescent staining were detected simultaneously.

### 3.4 Gluc activity accurately depict changes in LDLR expression

**FIGURE 3:**
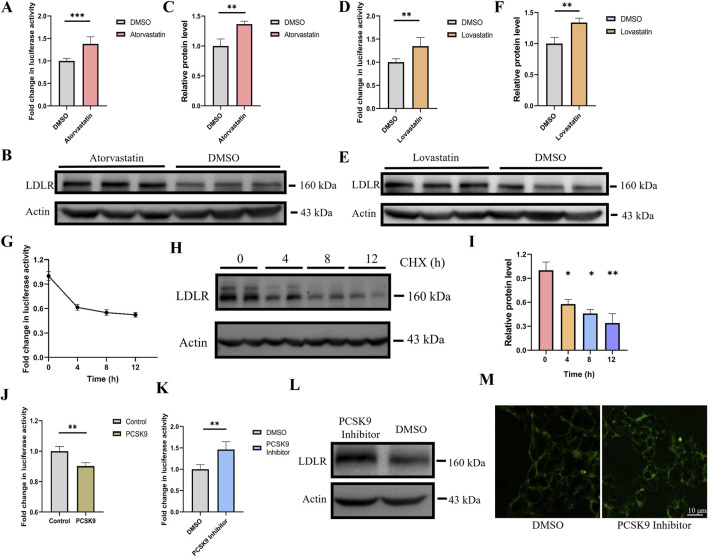
Gluc activity accurately depict changes in LDLR expression. **(A)** Luciferase activity was measured 24 h after treatment with 10 μM atorvastatin (n = 6). **(B, C)** LDLR protein expression was analyzed and quantified by Western blot after atorvastatin treatment (n = 3). **(D)** Luciferase activity was measured 24 h after treatment with 10 μM lovastatin (n = 6). **(E, F)** LDLR protein expression was analyzed and quantified by Western blot after lovastatin treatment (n = 3). **(G)** Cells were treated with 50 μg/mL CHX, and luciferase activity was measured at 0, 4, 8, and 12 h (n = 4). **(H, I)** After CHX treatment, LDLR protein expression was analyzed by Western blot at 0, 4, 8, and 12 h, and the relative protein levels were quantified (n = 2). **(J)** After transfecting PCSK9 for 48 h, luciferase activity was measured in the reporter gene cell line (n = 4). **(K)** Knock-in cell line was treated with PCSK9 inhibitor, luciferase activity was measured after 48 h (n = 4). **(L)** Knock-in cell line was treated with PCSK9 inhibitor, the expression level of LDLR in cell lysate as determined via Western blot **(M)** After treatment with the PCSK9 inhibitor, the fluorescence intensity of endogenous EGFP was analyzed using confocal microscopy in the knock-in cell line. *p < 0.05, **p < 0.01, ***p < 0.001 by Student’s t-test **(A, B, C, D, F, J and K)** or One-Way ANOVA **(I)**. Data represent the mean ± SEM.

We tested whether Gluc activity in the cell culture medium accurately reflects changes in endogenous LDLR expression. Statins are widely used clinical drugs for lowering cholesterol, with their core mechanism of action being the upregulation of LDLR expression on the surface of hepatocytes, which accelerates the clearance of LDL-C from the bloodstream. Statins competitively inhibit HMG-CoA reductase, the rate-limiting enzyme in cholesterol synthesis, thereby reducing the intracellular levels of mevalonate and inhibiting *de novo* cholesterol synthesis (Slater and MacDonald, 1988; Corsini et al., 1995; Istvan and Deisenhofer, 2001). The resulting cholesterol deficiency releases the inhibition of SREBP, which activates the transcription of several target genes, including LDLR, leading to increased expression of LDLR. To demonstrate the practical applicability of our dual-reporter system in drug discovery, we treated reporter cells with atorvastatin and lovastatin, and measured Gluc activity in the culture medium. We found that treatment with atorvastatin and lovastatin resulted in a 37% and 35% increase in Gluc activity, respectively (Figures 3A–C). Additionally, LDLR expression in the cell lysates was assessed via Western blot analysis, showing a 36% and 33% increase in LDLR expression, respectively (Figures 3D–F).

Cycloheximide (CHX), which inhibits eukaryotic protein synthesis by blocking translation elongation (Schneider-Poetsch et al., 2010), was used to treat the reporter cells. Gluc activity was measured at 0, 4, 8, and 12 h, showing decreases of approximately 40%, 45%, and 50% at the respective time points (Figure 3G). In parallel, Western blot analysis of the cell lysates revealed that LDLR protein expression decreased by approximately 40%, 50%, and 60% at the corresponding time intervals (Figures 3H, I). The trend observed in the Gluc activity data closely mirrored the changes in LDLR protein levels, indicating that the reporter gene cell line accurately reflects alterations in LDLR protein expression.

Proprotein convertase subtilisin/kexin type 9 (PCSK9) promotes the degradation of LDLR by the binding of its catalytic domain to the N-terminal of the epidermal growth factor-like repeat A (EGF-A) domain of LDLR on the cell membrane (Zhang et al., 2007; Seidah and Prat, 2012). PCSK9 inhibitors function by specifically binding to PCSK9, thereby blocking its interaction with LDLR, which disrupts the endocytosis and degradation of LDLR (Horton et al., 2007). We overexpressed PCSK9 in the reporter gene cell line and observed that PCSK9 overexpression reduced the Gluc activity in the reporter cell line (Figure 3J). Furthermore, the reporter cells were treated with a PCSK9 inhibitor for 48 h, and the expression of both the Gluc reporter and LDLR was compared. Luciferase activity in the supernatant and LDLR expression in the cell lysate, as determined by Western blot, were upregulated with similar dynamics, reaching approximately 1.4-fold induction at 48 h post-PCSK9 inhibitor treatment (Figures 3K, L). Confocal microscopy analysis revealed that, following PCSK9 inhibitor treatment, the fluorescence intensity of EGFP was also increased (Figure 3M). Notably, Gluc activity in the supernatants was very stable, and the supernatants could be stored at room temperature for several days without a significant loss of luciferase activity.

### 3.5 Gluc activity as a tool for screening transcription factors regulating LDLR expression

**FIGURE 4:**
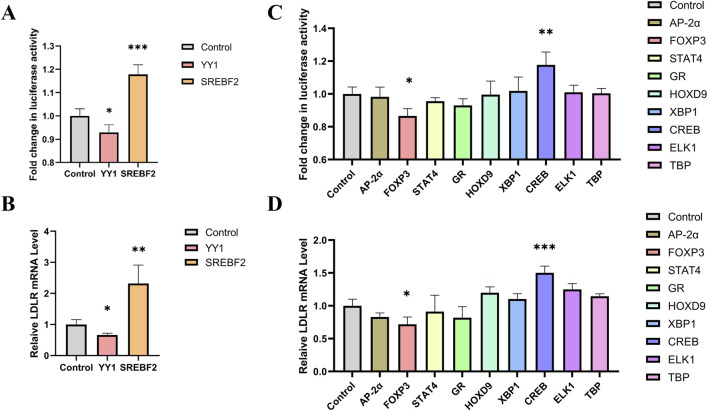
Screening of transcription factors for regulating LDLR expression. **(A, B)** Knock-in cell line was transfected with YY1 and SREBF2, respectively, and luciferase activity and LDLR mRNA level were measured after 48 h (n = 4). **(C)** Luciferase activity in the reporter gene cell line 48 h after transfection with transcription factors AP-2α, FOXP3, STAT4, GR, HOXP9, XBP1, CREB, ELK1 and TBP (n = 4). **(D)** Relative expression levels of LDLR mRNA following transfection with the transcription factors (n = 4). *p < 0.05, **p < 0.01, ***p < 0.001 by One-Way ANOVA. Data represent the mean ± SEM.

Sterol regulatory element-binding protein 2 (SREBP-2) has been previously reported to activate LDLR transcription, while Yin Yang 1 (YY1) has been shown to alternatively repress LDLR expression (Rice et al., 2014; Qian and Zhao, 2022). We tested these transcription factors in our reporter cell line and found that YY1 significantly reduced luciferase activity, while SREBF2 increased luciferase activity (Figure 4A). Additionally, the expression level of LDLR mRNA showed consistent changes (Figure 4B). These results indicate that the knock-in reporter system is an effective and convenient tool for investigating transcription factors.

**FIGURE 5:**
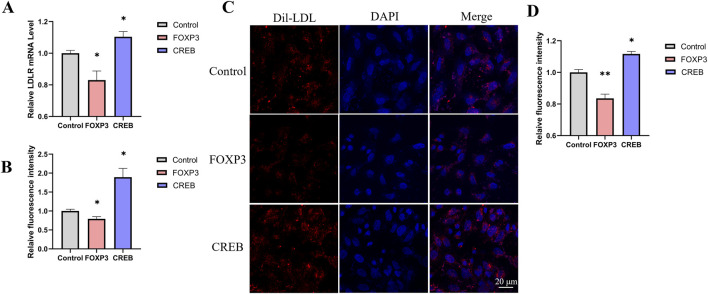
The effect of transcription factors on DiI-LDL uptake in HepG2 cells. **(A)** Changes in LDLR relative expression levels 48 h after transfection with FOXP3 or CREB in HepG2 cells (n = 4). **(B)** Relative fluorescence intensity of DiI-LDL measured by a microplate reader 48 h after transfection with FOXP3 or CREB in HepG2 cells (n = 4). **(C, D)** Confocal microscopy images showing the uptake of DiI-LDL by HepG2 cells transfected with FOXP3 or CREB, with quantification of DiI fluorescence intensity per cell using ImageJ (D, n = 3). *p < 0.05, **p < 0.01, ***p < 0.001 by One-Way ANOVA. Data represent the mean ± SEM. Scale bar, 20 μm.

To screen for transcription factors that may regulate LDLR expression, we analyzed the promoter region of the LDLR gene using the PROMO website. Nine transcription factors were selected for further investigation: AP-2α, FOXP3, STAT4, GR, HOXP9, XBP1, CREB, ELK1, and TBP. These transcription factors were individually cloned into the pcDNA3.1 vector to construct overexpression plasmids, which were then transfected into the reporter cell line. Luciferase activity was measured 48 h post-transfection. We observed that FOXP3 significantly decreased luciferase activity, while CREB increased it (Figure 4C). Additionally, we measured LDLR mRNA level and found that overexpression of FOXP3 and CREB led to changes in LDLR expression that were consistent with the luciferase activity results (Figure 4D). The regulation of LDLR expression by FOXP3 and CREB was further confirmed in HepG2 cells, with FOXP3 decreasing and CREB increasing LDLR expression (Figures 5A, B), as well as the corresponding DiI-LDL uptake (Figures 5C, D).

### 3.6 SREBF2 mediates the CREB-induced regulation of LDLR expression

**FIGURE 6:**
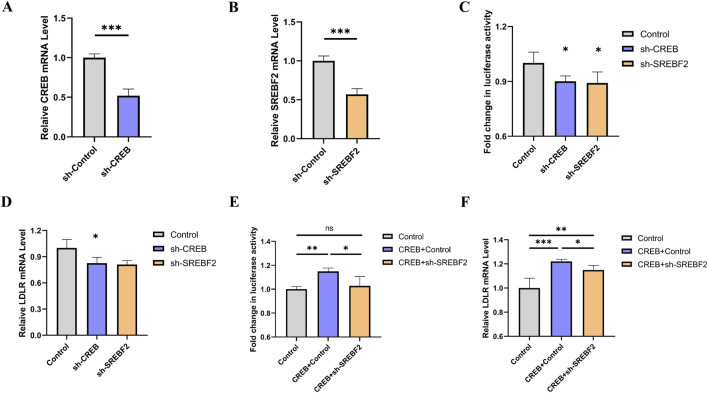
Knockdown of CREB or SREBF2 reduces LDLR expression in reporter gene cell line. **(A, B)** Relative expression levels of CREB and SREBF2 in reporter gene cell line 48 h after transfection with sh-CREB or sh-SREBF2 plasmids, respectively (n = 4). **(C, D)** Luciferase activity of reporter genes and relative expression levels of LDLR 48 h after transfection with sh-CREB or sh-SREBF2 (n = 4). **(E, F)** In reporter gene cell line, overexpression of CREB combined with sh-SREBF2 transfection showed that knockdown of SREBF2 attenuated the CREB-induced increase in luciferase activity and concurrently reversed the expression levels of LDLR (n = 4). *p < 0.05, **p < 0.01, ***p < 0.001 by Student’s t-test **(A, B)** or One-Way ANOVA **(C–F)**. Data represent the mean ± SEM.

To investigate whether SREBF2 is involved in the regulation of LDLR by CREB, we constructed knockdown plasmids targeting CREB and SREBF2, respectively. Upon transfection of these plasmids into the reporter gene cell line, a significant knockdown effect was observed (Figures 6A, B). After 48 h of transfection, luciferase activity was measured, revealing a marked reduction in luciferase activity following knockdown (Figure 6C). Concurrently, the relative expression level of LDLR in the reporter gene cell line was assessed using qRT-PCR, which also showed a significant decrease in LDLR expression (Figure 6D). To further explore whether the regulation of LDLR by CREB occurs via the SREBF2 pathway, we overexpressed CREB while simultaneously knocking down SREBF2 in the reporter gene cell line. We found that knocking down SREBF2 abrogated the effect of CREB overexpression on luciferase activity (Figure 6E). At the mRNA level, SREBF2 knockdown similarly mitigated the effect of CREB overexpression on LDLR mRNA (Figure 6F). Based on these results, we infer that SREBF2 is involved in the regulation of LDLR expression by CREB.

**FIGURE 7:**
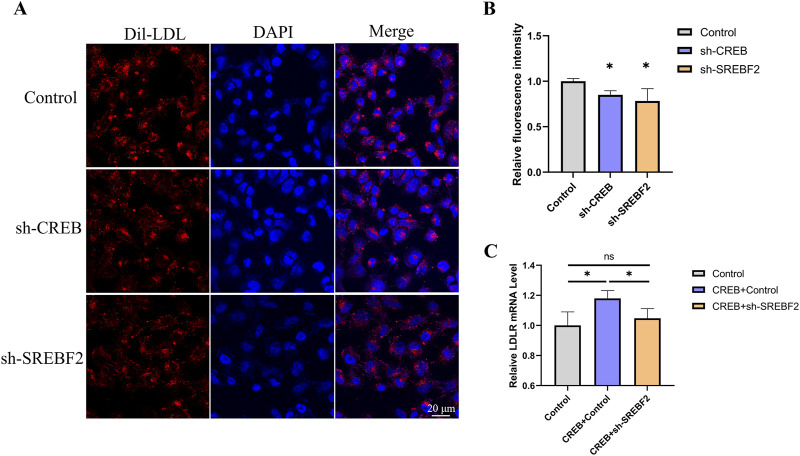
Knockdown of CREB or SREBF2 reduces Dil-LDL uptake in HepG2 cells. **(A, B)** Uptake of Dil-LDL in HepG2 cells 48 h after transfection with sh-CREB or sh-SREBF2, observed by confocal microscopy and quantified using ImageJ to measure the relative fluorescence intensity of Dil in each cell (n = 3). **(C)** Overexpression of CREB in HepG2 cells followed by transfection with sh-SREBF2; knockdown of SREBF2 reduced the effect of CREB on LDLR expression in HepG2 cells (n = 4). *p < 0.05, **p < 0.01, ***p < 0.001 by One-Way ANOVA. Data represent the mean ± SEM. Scale bar, 20 μm.

We next examined whether knockdown of CREB or SREBF2 affects the uptake of Dil-LDL in HepG2 cells. After transfecting HepG2 cells with sh-CREB or sh-SREBF2 for 48 h, the culture medium was refreshed, and Dil-LDL was added. The uptake of LDL was observed using confocal microscopy, and the fluorescence intensity of each cell was quantified. We found that knockdown of CREB or SREBF2 both significantly reduced the uptake of Dil-LDL in HepG2 cells (Figures 7A, B). Subsequently, we overexpressed CREB while knocking down SREBF2 in HepG2 cells to investigate whether interference with SREBF2 would diminish CREB’s effect on LDLR expression. The results in HepG2 cells were consistent with those observed in reporter gene cell line: knockdown of SREBF2 reversed the CREB-induced increase in LDLR expression, further indicating that SREBF2 plays a role in the regulation of LDLR expression by CREB (Figure 7C).

## 4 Discussion

LDLR is a principal target for novel therapeutics aimed at metabolic risk factors and diseases. Although current lipid-lowering medications, such as statins, are highly effective in reducing serum LDL levels in the general population, a significant number of patients with extremely elevated LDL levels exhibit either no response or only a moderate response (Defesche et al., 2017; Brandts and Ray, 2021). As a novel therapeutic target for hyperlipidemia, a pressing challenge is how to maintain high expression levels of LDLR to enhance the clearance of LDL from the serum.

To facilitate high-throughput screening of small-molecule drugs targeting the LDLR gene and to investigate its transcriptional regulation, this study employed the CRISPR/Cas9 system with both positive and negative selection to construct a luciferase reporter system, specifically the LDLR-EGFP-Gluc knock-in HEK293 cell line. The construction of this cell line was confirmed through methods including electrophoresis band analysis, confocal microscopy for EGFP expression, and genomic sequencing. In this cell line, an EGFP-Gluc cassette was integrated into the genome under the control of the endogenous LDLR promoter. The intensity of EGFP fluorescence and the luciferase secreted by the cell line accurately reflect the expression of endogenous LDLR, thereby greatly facilitating the study of LDLR gene function.

Following the successful construction of the cell line, we treated the cells with statins, CHX and PCSK9 inhibitor to demonstrate that the reporter system accurately reflects changes in endogenous LDLR expression. In previous studies, the effects of PCSK9 inhibitors on LDLR expression (Cameron et al., 2006; Qian Y.-W. et al., 2007; Wang et al., 2020) and CHX-induced LDLR protein degradation (Makar et al., 1994; Kizhakkedath et al., 2018) were primarily quantified using traditional methods such as Western blotting. However, these approaches do not allow for real-time, dynamic characterization of LDLR expression changes. Using our reporter cell line, the observed trends in LDLR expression were consistent with those reported in previous studies. Moreover, our system enables real-time, visualized monitoring of LDLR dynamics, providing a more comprehensive and immediate assessment of its regulation. Furthermore, a key advantage of our screening assay is that LDLR expression can be directly quantified through Gluc activity in the supernatant without requiring cell lysis. This feature significantly simplifies sample analysis and enzymatic activity detection, making the assay more convenient for monitoring reporter gene expression over time and facilitating high-throughput screening applications.

Comparing LDLR expression (both mRNA and protein levels), EGFP intensity, luciferase activity, and DiI-LDL intensity is crucial for validating whether our dual-reporter system functions as intended. We measured LDLR protein expression via Western blot analysis following treatment with atorvastatin and lovastatin. The expression of LDLR increased by 36% and 33%, respectively, while Gluc activity in the culture medium increased by 37% and 35%, respectively. These findings indicate that Gluc activity and Western blot analysis can similarly capture trends in LDLR expression. After treating cells with a PCSK9 inhibitor, Gluc activity increased by 46%, LDLR protein expression rose by 42%, and EGFP intensity increased by 29%. These trends were consistent, demonstrating that luciferase activity, Western blot analysis, and EGFP intensity can all be used to assess changes in LDLR expression. When we overexpressed the transcription factors FOXP3 and CREB, the LDLR mRNA levels decreased by 17% and increased by 10%, respectively. Similarly, DiI-LDL intensity decreased by 16% and increased by 12%. The trends were consistent, suggesting that both mRNA expression and DiI-LDL intensity can reliably reflect changes in LDLR expression. Although the values from these different measurements do not perfectly align, the trends observed in LDLR expression, EGFP intensity, luciferase activity, and DiI-LDL intensity are consistent. This further supports the idea that our dual-reporter system, through luciferase activity and EGFP intensity, can effectively represent LDLR expression.

LDLR expression is regulated by various transcription factors. Therefore, we employed the known transcription factors YY1 and SREBF2, which regulate LDLR expression, to verify the reliability of the reporter system. The results showed that changes in luciferase activity were consistent with changes in LDLR mRNA level, indicating that this cell line can be used to screen for transcription factors that regulate LDLR expression. Using the reporter gene cell line, we found that FOXP3 and CREB significantly regulated LDLR expression. We further tested their effects on LDLR expression and Dil-LDL uptake in HepG2 cells. Consistent with the reporter gene cell line results, LDLR mRNA level was decreased by FOXP3 and increased by CREB in HepG2 cells, which corresponded to reduced and enhanced Dil-LDL uptake, respectively. FOXP3, a member of the forkhead transcription factor family, has been identified as a master regulator in the development and function of regulatory T cells (Tregs) (Schubert et al., 2001; Fontenot et al., 2003). Recent reports suggest that FOXP3 is also expressed in normal non-lymphoid cells or cancer cells, indicating that the transcriptional regulatory role of FOXP3 may be broader than initially thought (Martin et al., 2010). Our results suggest that FOXP3 may be involved in the regulation of LDLR expression.

CREB is localized in the nucleus and acts as a transcription factor, once CREB is activated and CREB-binding protein (CBP) is recruited, transcription is initiated (Dyson and Wright, 2016). There have been few reports on the regulation of LDLR by CREB, but some researchers believe that CREB can directly bind to the sterol-independent regulatory element (SIRE) region of LDLR (Liu et al., 2000). However, whether CREB regulates LDLR expression through other pathways remains to be explored. SREBF2 is a key regulator in maintaining cholesterol homeostasis and can regulate the expression of the LDLR gene (Brown and Goldstein, 1997; Sun et al., 2007). Studies have shown that the putative activation domain of SREBP specifically binds to the amino-terminal domains of CREB-binding protein, and CREB enhances the ability of SREBP to activate the transcription of reporter genes in HeLa cells (Oliner et al., 1996). To investigate whether CREB could regulate LDLR through the SREBP2 pathway, we overexpressed CREB and simultaneously knocked down SREBF2, finding that knockdown of SREBF2 counteracted the effects of CREB overexpression on the expression of luciferase and LDLR mRNA in the reporter gene system. The same experimental results were confirmed in the HepG2 cell line. Based on these results, we hypothesize that CREB may regulate LDLR expression not only directly but also indirectly by regulating the expression of SREBP2.

In summary, this study successfully developed a luciferase reporter system based on the endogenous LDLR promoter in HEK293 cells. Demonstrates that the reporter gene cell line can reflect LDLR expression by measuring luciferase activity in the culture medium supernatant. The accuracy of this system was further validated using known transcription factors and small molecule inhibitors. Using this system, we screened transcription factors that regulate LDLR expression, discovering that FOXP3 may be involved in the regulation of LDLR expression, and CREB may regulate LDLR expression through both direct and indirect mechanisms. Given the role of LDLR in many lipid metabolism processes, this novel system may aid in the screening of small molecule drugs or transcription factors that target LDLR expression, as well as in studies related to LDLR’s physiological functions.

## Data Availability

The original contributions presented in the study are included in the article/Supplementary Material, further inquiries can be directed to the corresponding author.
