# Supplementary material for: A dual-reporter LDLR system integrating fluorescence and luminescence for understanding LDLR regulation and facilitating drug discovery
Source: Front Mol Biosci. 2025 Mar 13;12:1552085. doi: 10.3389/fmolb.2025.1552085 (PMC11966430; doi:10.3389/fmolb.2025.1552085)

Figure 1B: T7E1 assay to detect the cutting efficiency of sgRNA.

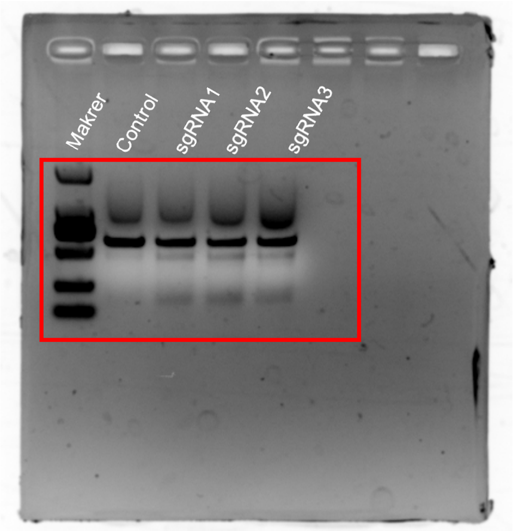

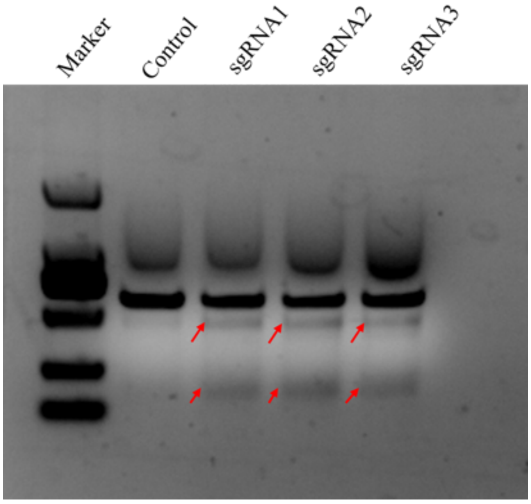

Original image

Figure 1B

Figure 1D: PCR detection of donor insertion in sorted single-cell clones.

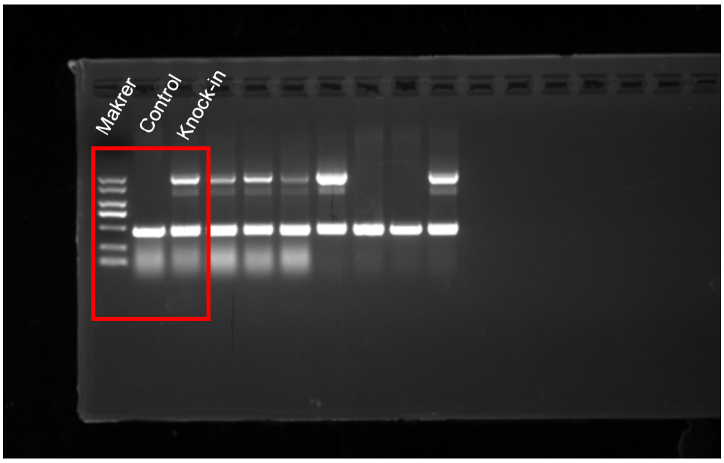

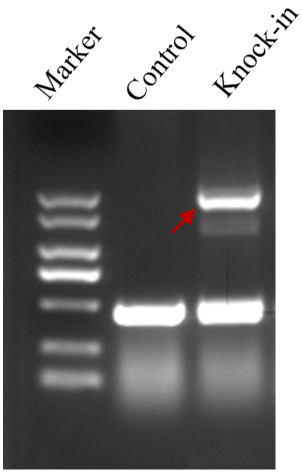

Original image

Figure 1D

Figure 2A: Cell morphology of wild-type HEK293 and knock-in cell line.

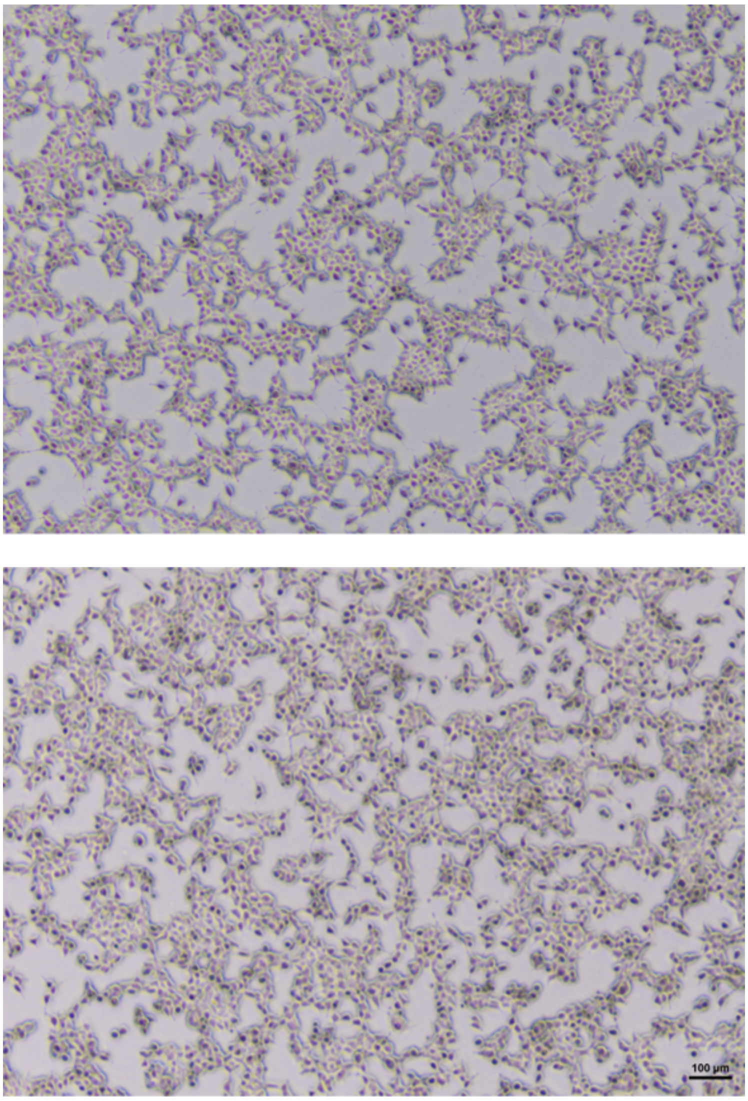

Figure 2B: LDL particle uptake was observed using confocal microscopy.

Control

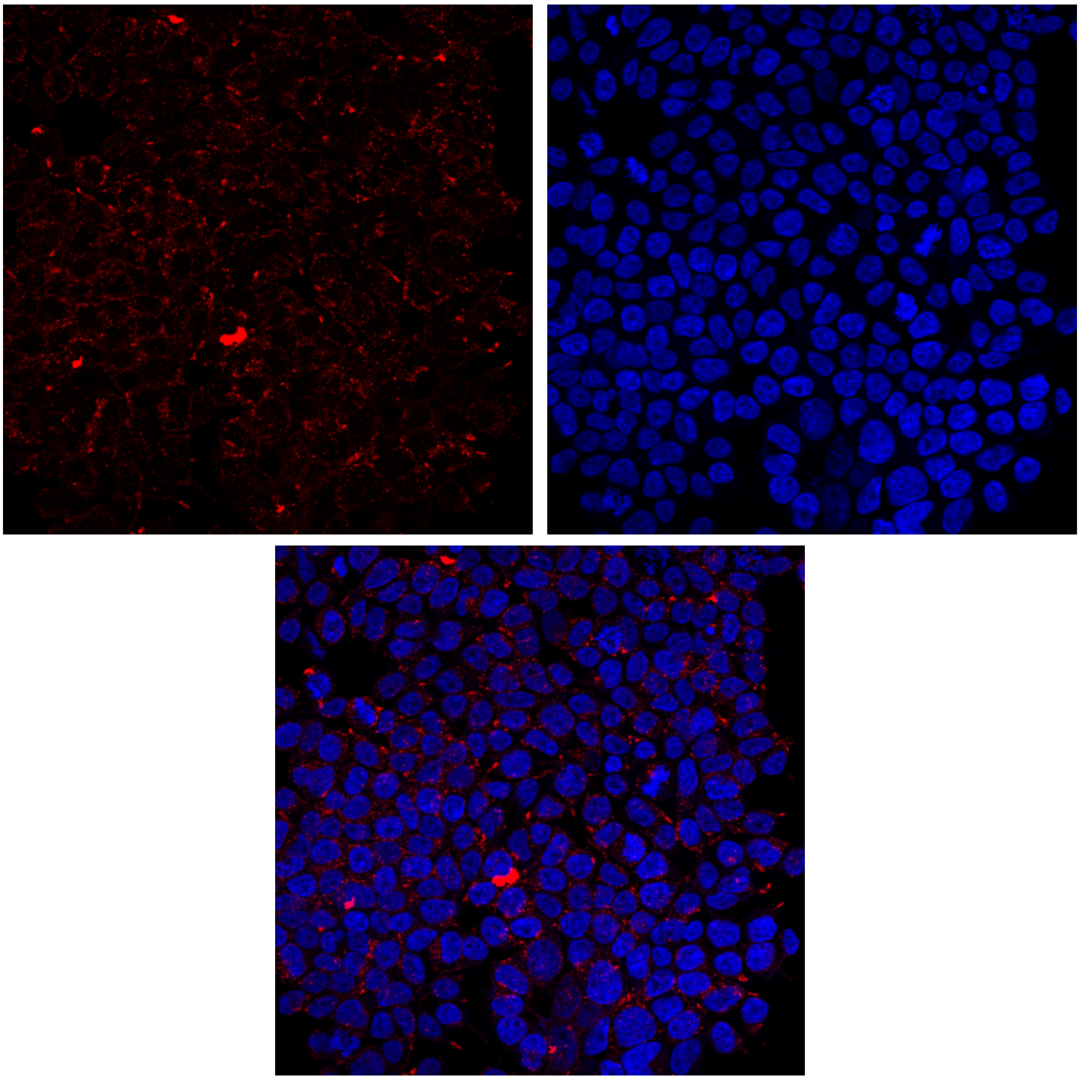

Knock-in

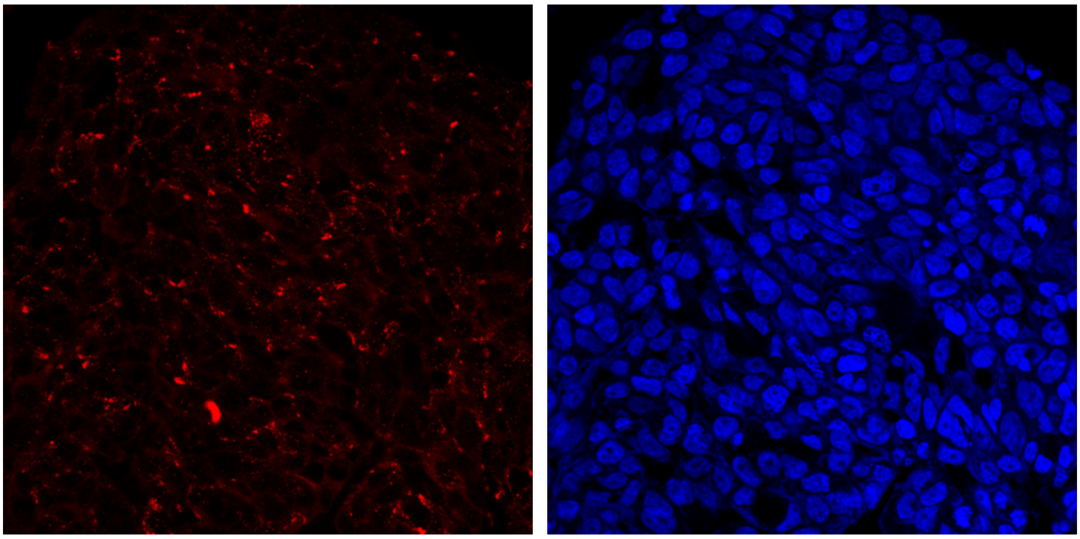

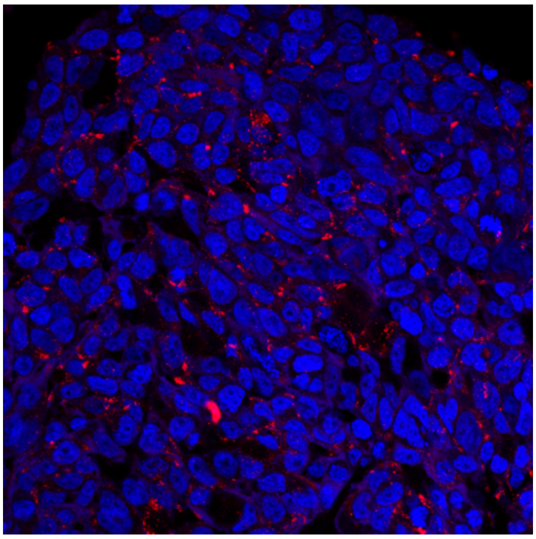

Figure 2E: Western blot analysis was performed to detect the expression of Gluc, LDLR, and LDLR-EGFP fusion protein in the knock-in cell line, using anti-Gluc and anti-LDLR antibodies.

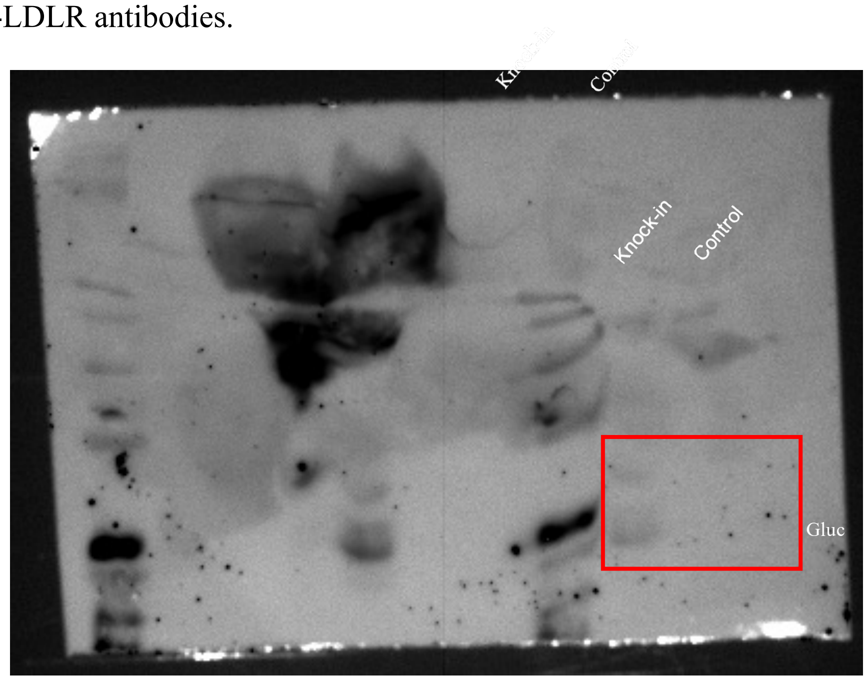

Original image

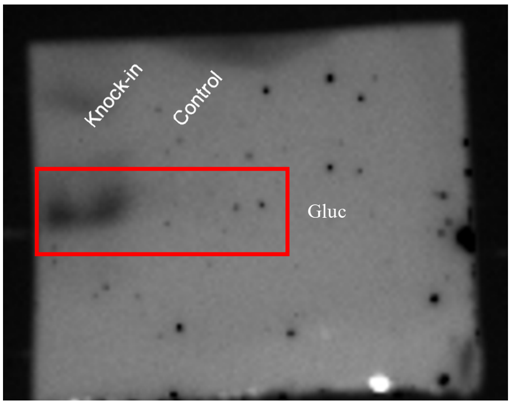

Original image (Second exposure)

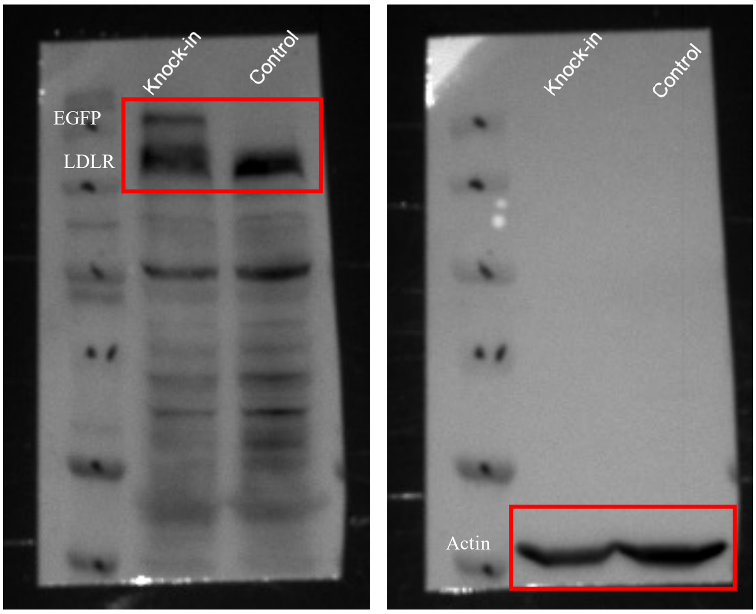

Original image

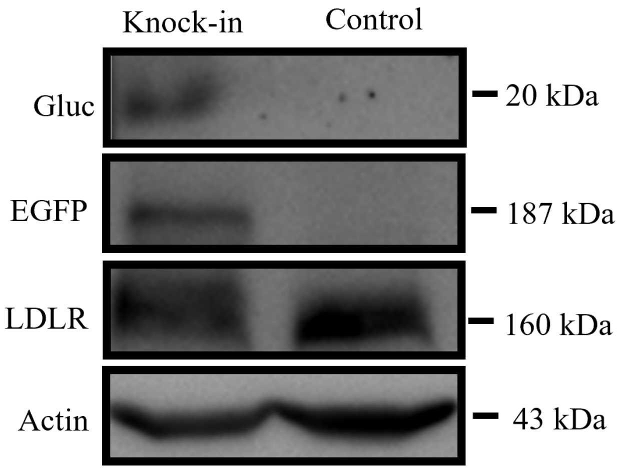

Figure 2E

Figure 2F: Expression of EGFP in the knock-in cell line observed by confocal microscopy.

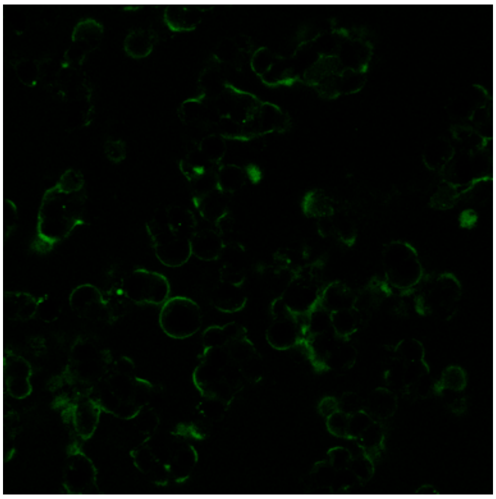

Figure 2G: EGFP fluorescence and LDLR fluorescent staining were detected by immunofluorescence staining.

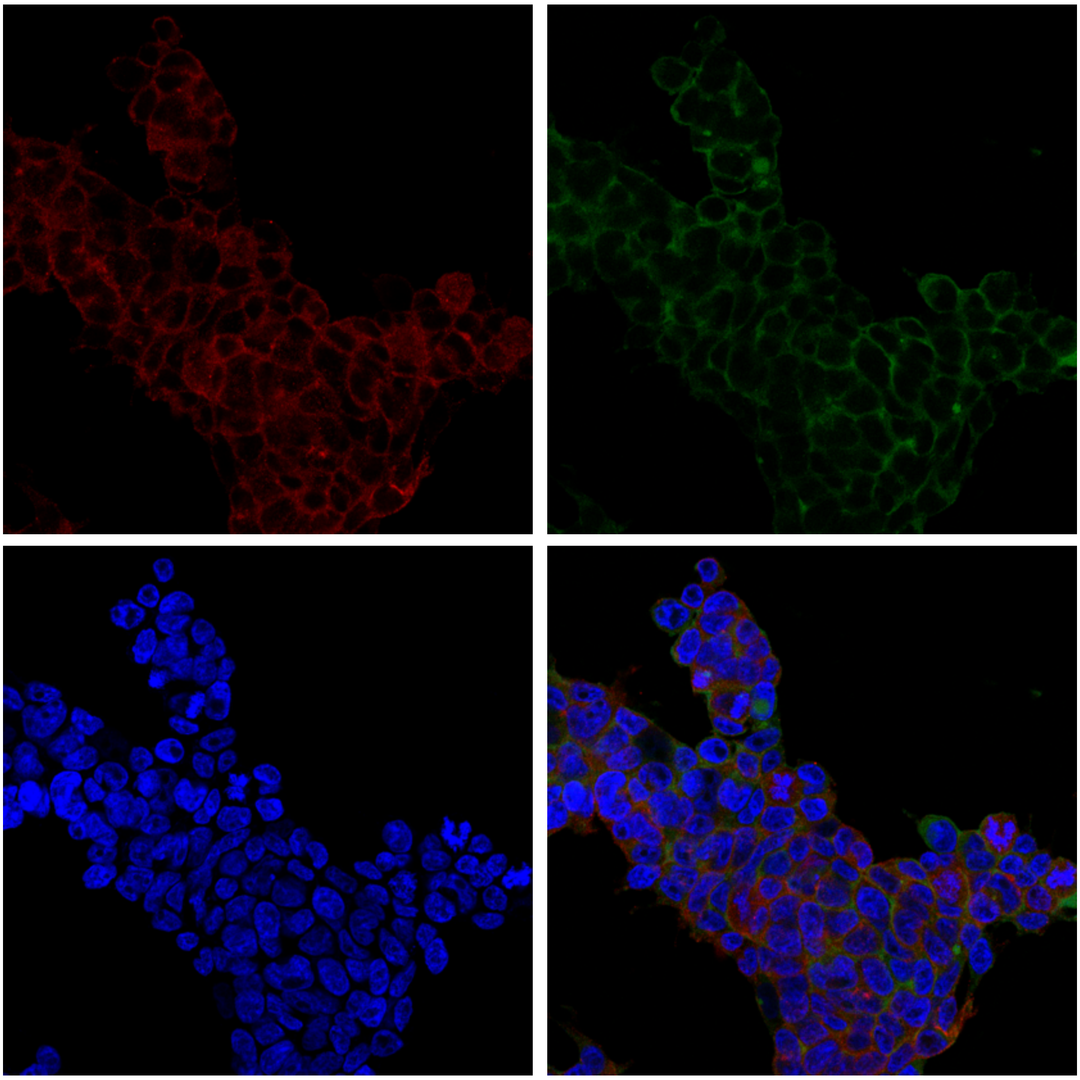

Figure 3B LDLR protein expression was analyzed by Western blot after atorvastatin

treatment.

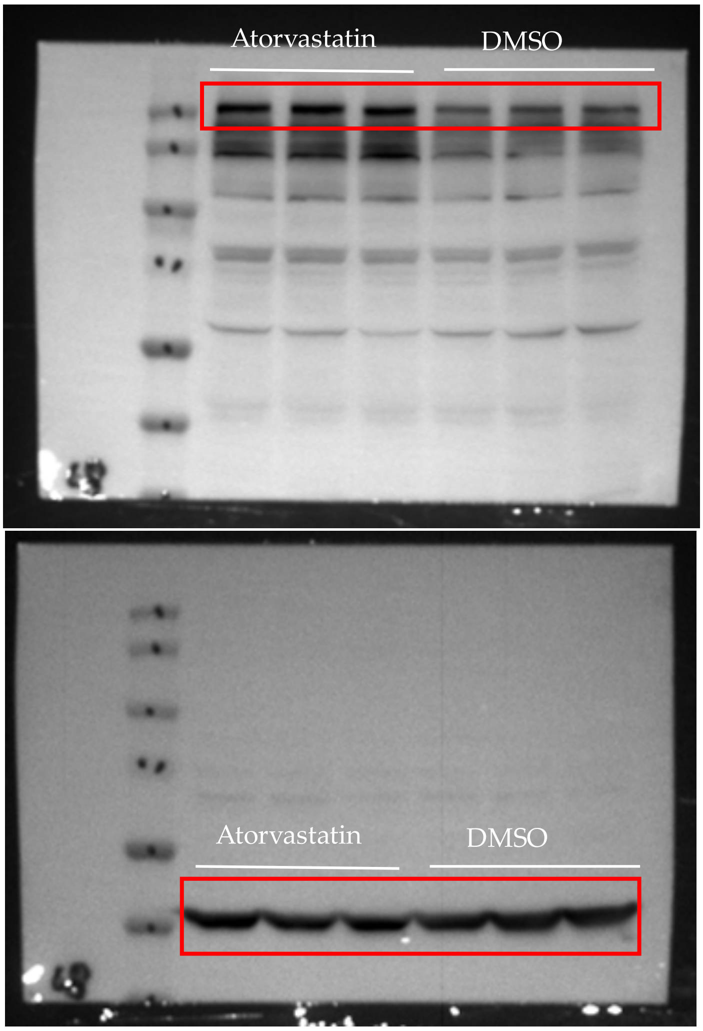

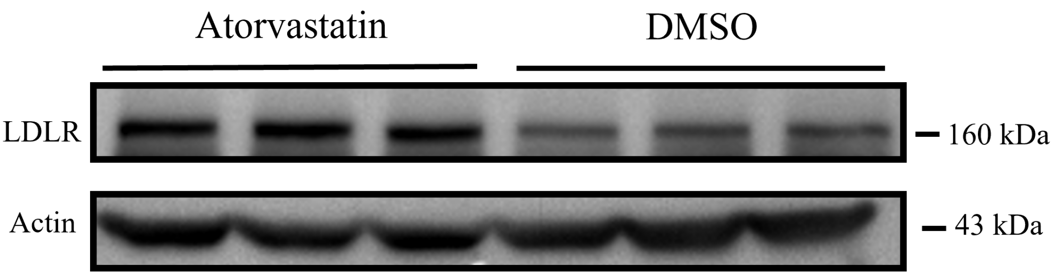

Figure 3B

Figure 3E LDLR protein expression was analyzed by Western blot after lovastatin treatment.

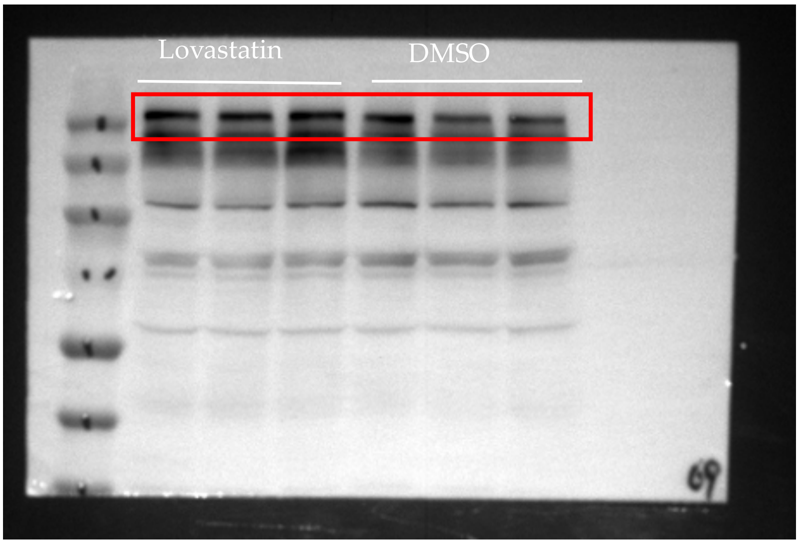

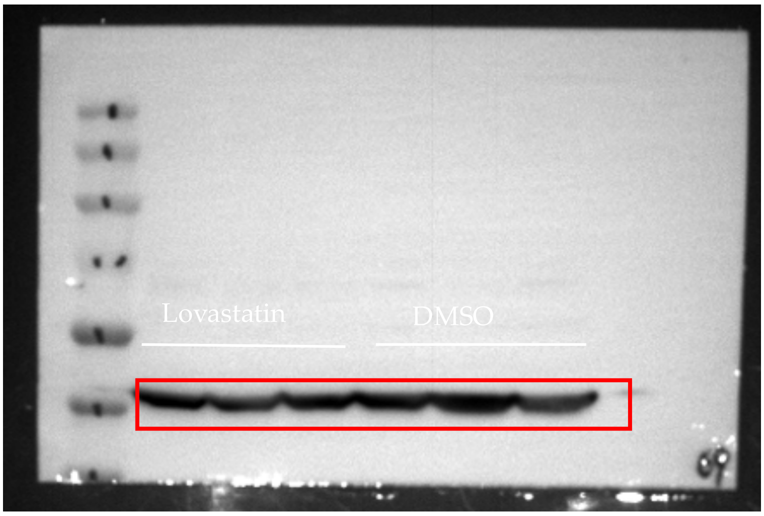

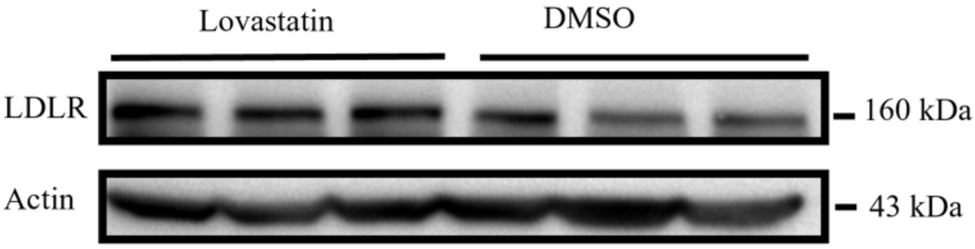

Figure 3E

Figure 3H: After CHX treatment, LDLR protein expression was analyzed by western blot.

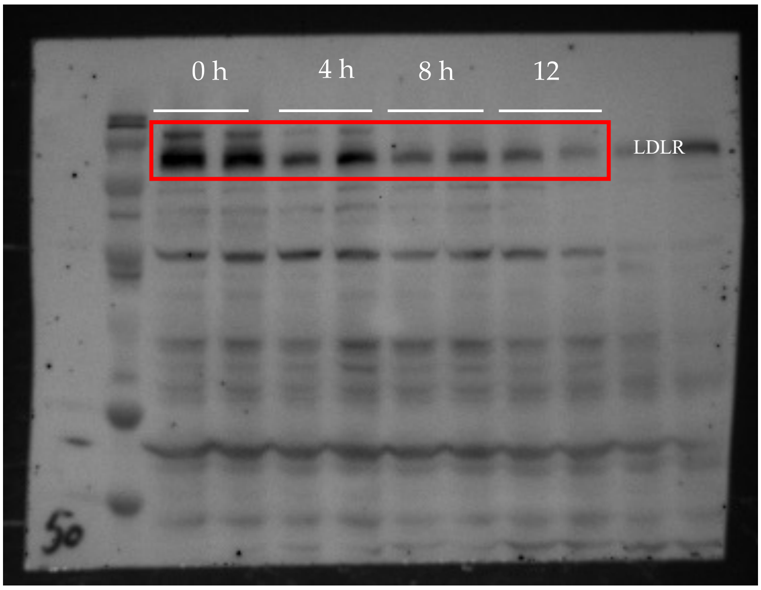

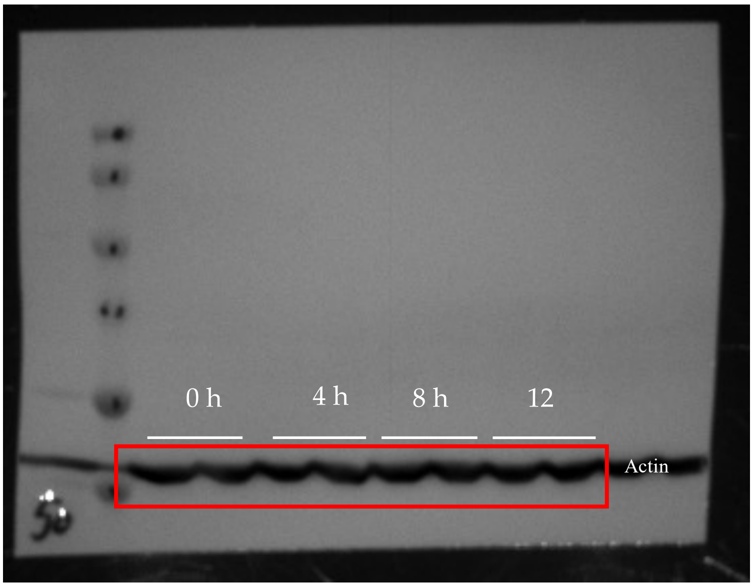

Original image

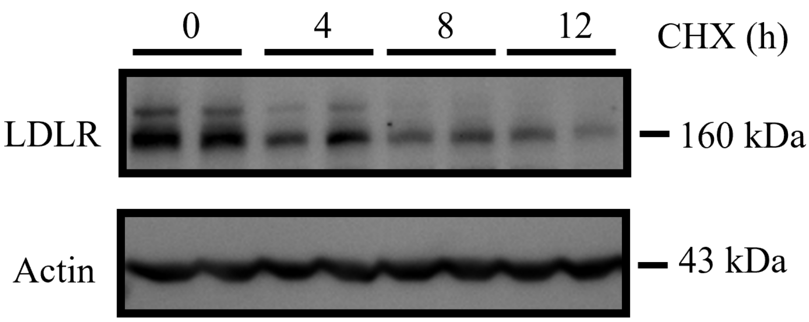

Figure 3H

Figure 3L: After treated with PCSK9 inhibitor, the expression level of LDLR in cell lysate as determined via western blot.

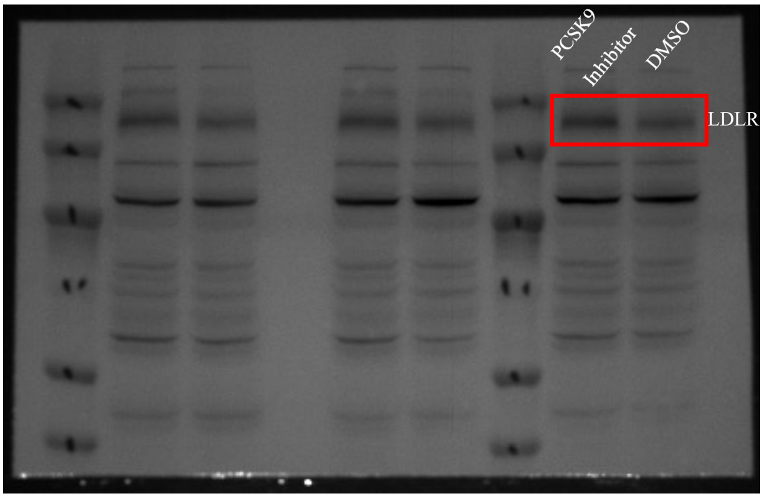

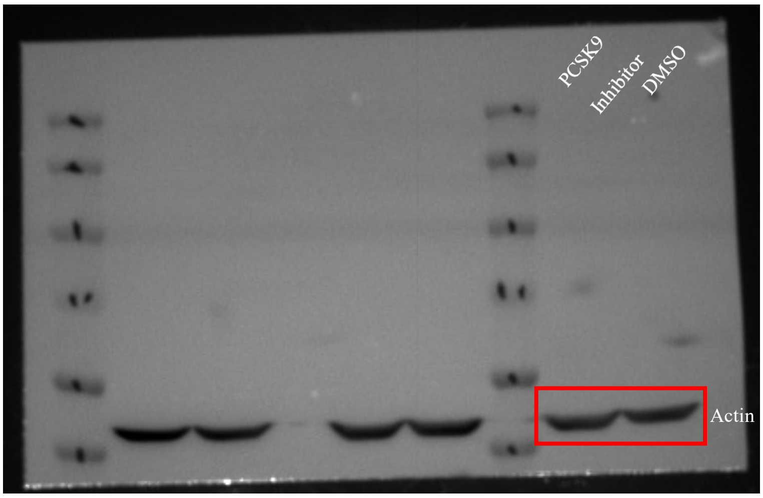

Original image

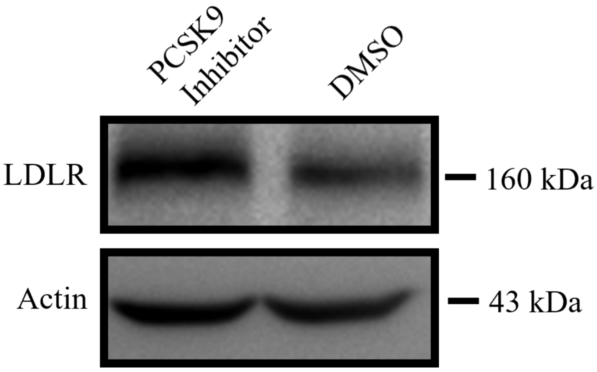

Figure 3H

Figure 3M: After treatment with the PCSK9 inhibitor, the fluorescence intensity of endogenous EGFP was revealed by confocal microscopy.

DMSO PCSK9 Inhibitor

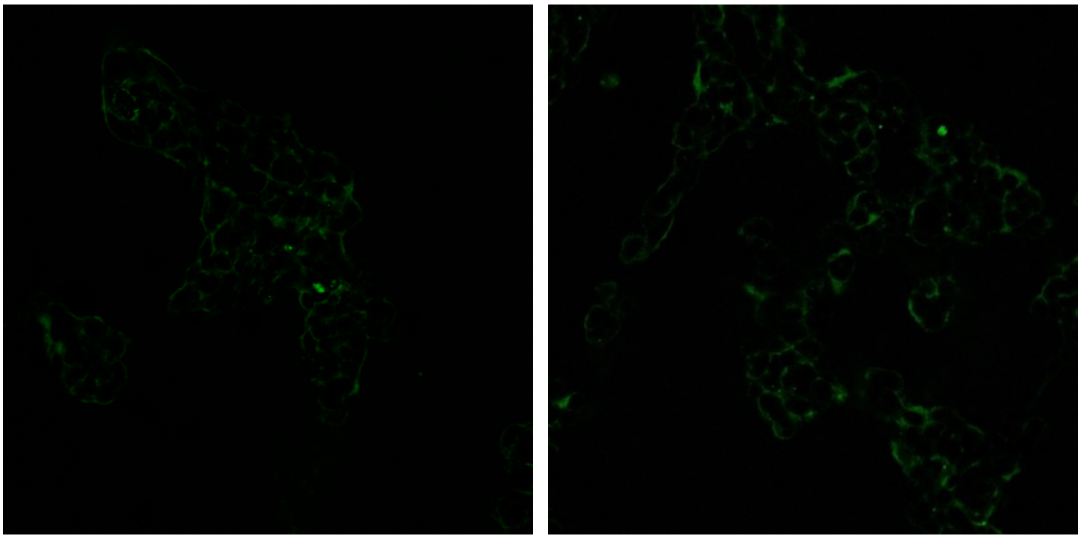

Figure 5C: Confocal microscopy images showing the uptake of DiI-LDL by HepG2 cells transfected with FOXP3 or CREB.

Control

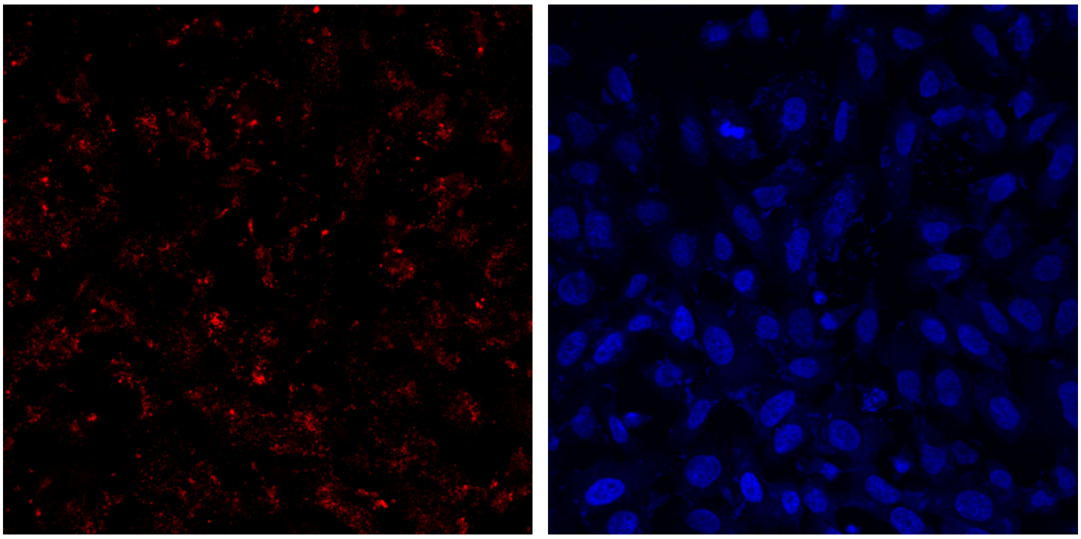

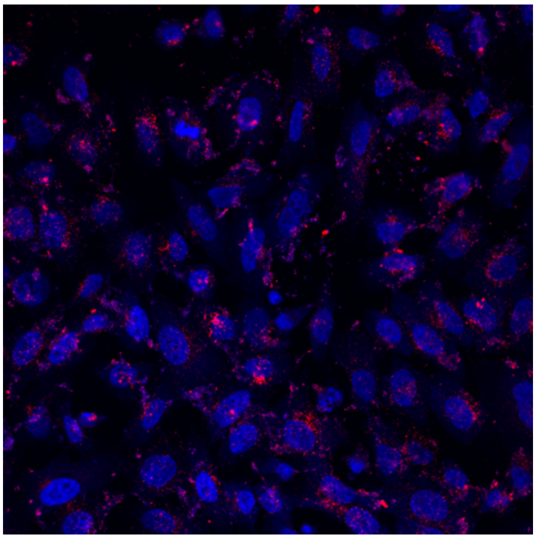

FOXP3

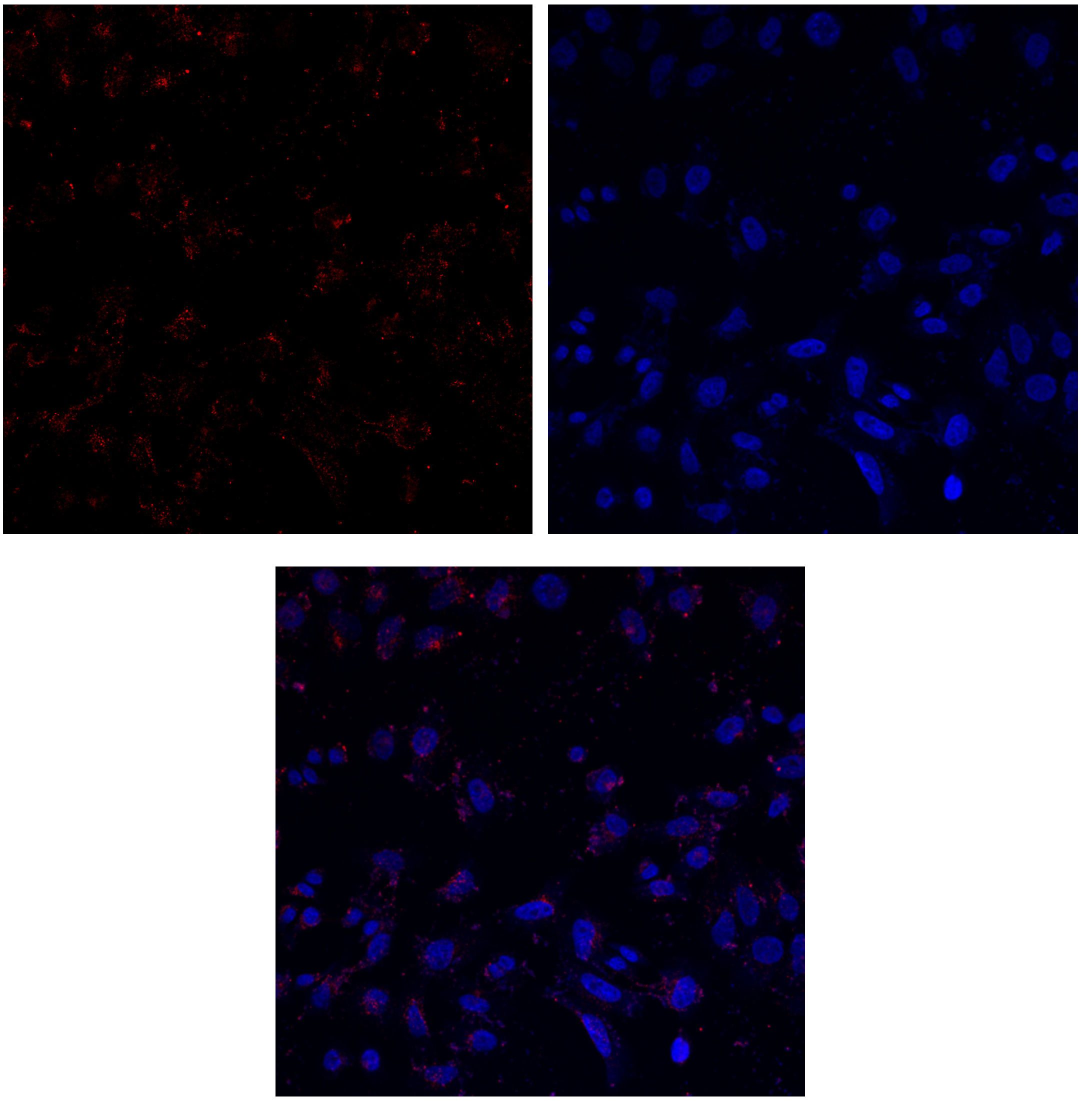

CREB

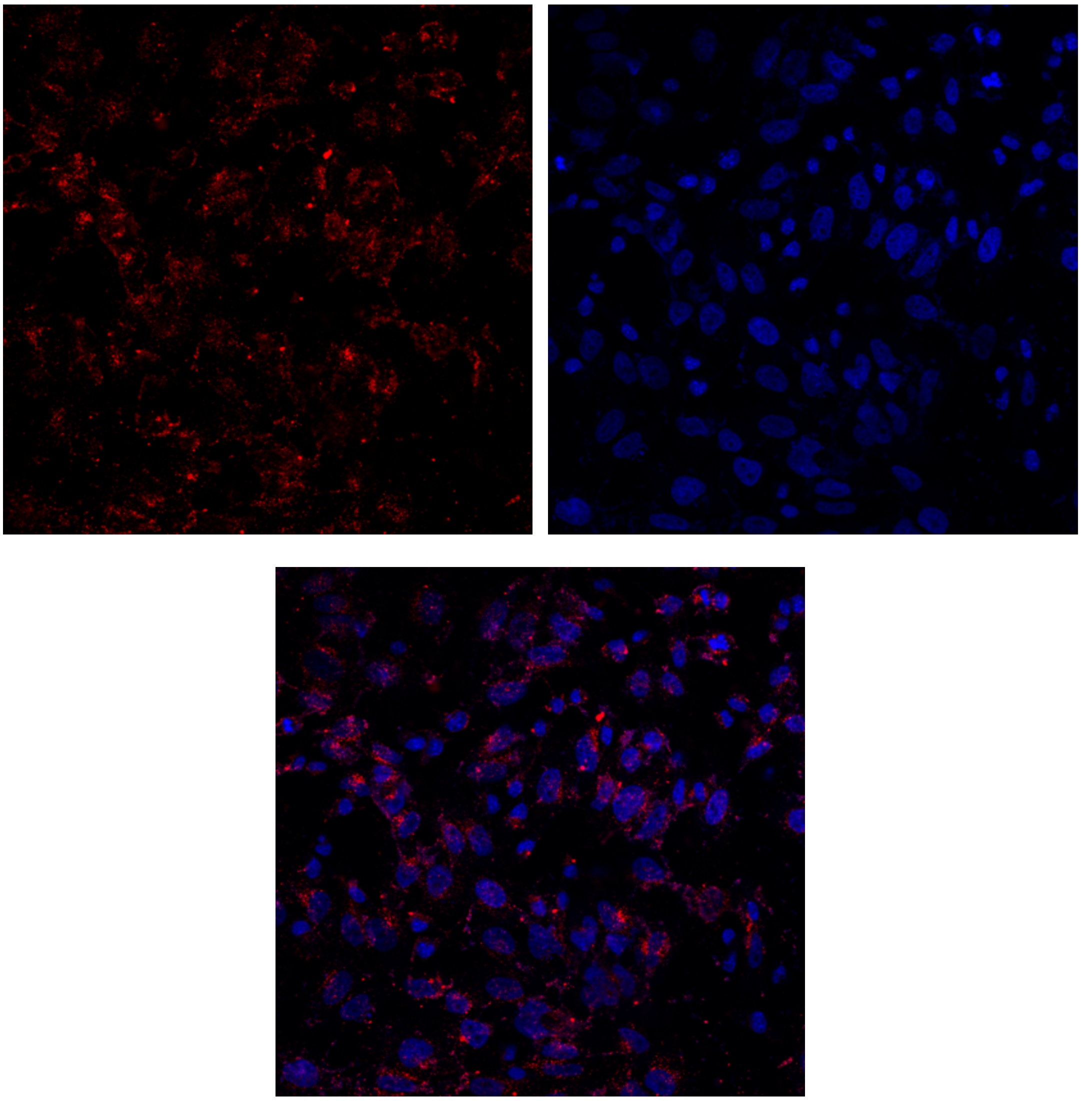

Figure 7A: Uptake of Dil-LDL in HepG2 cells 48 hours after transfection with sh-CREB or sh-SREBF2.

Control

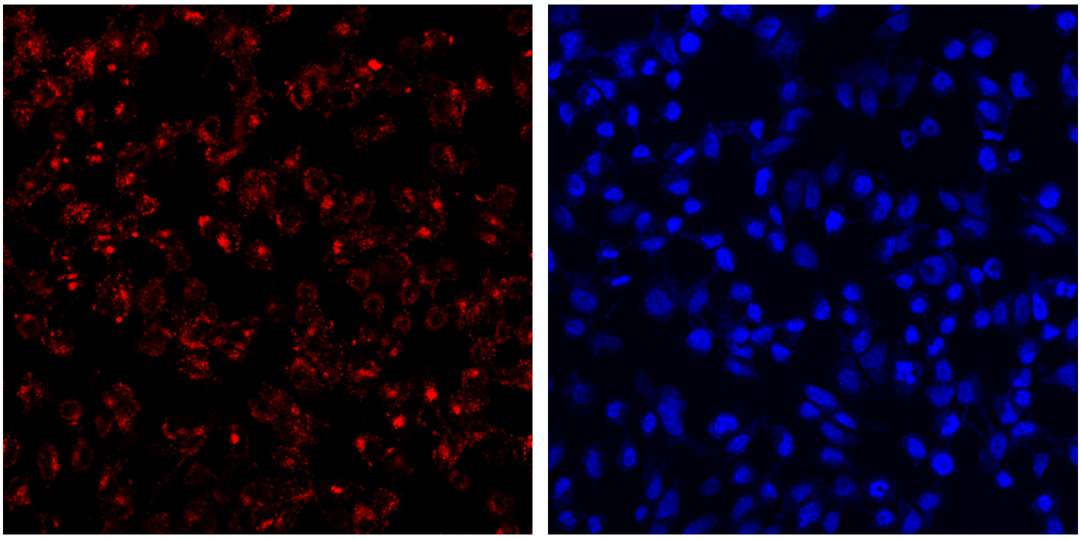

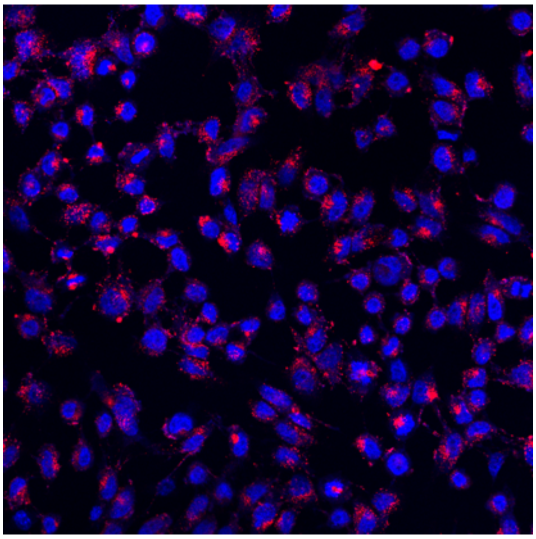

sh-CREB

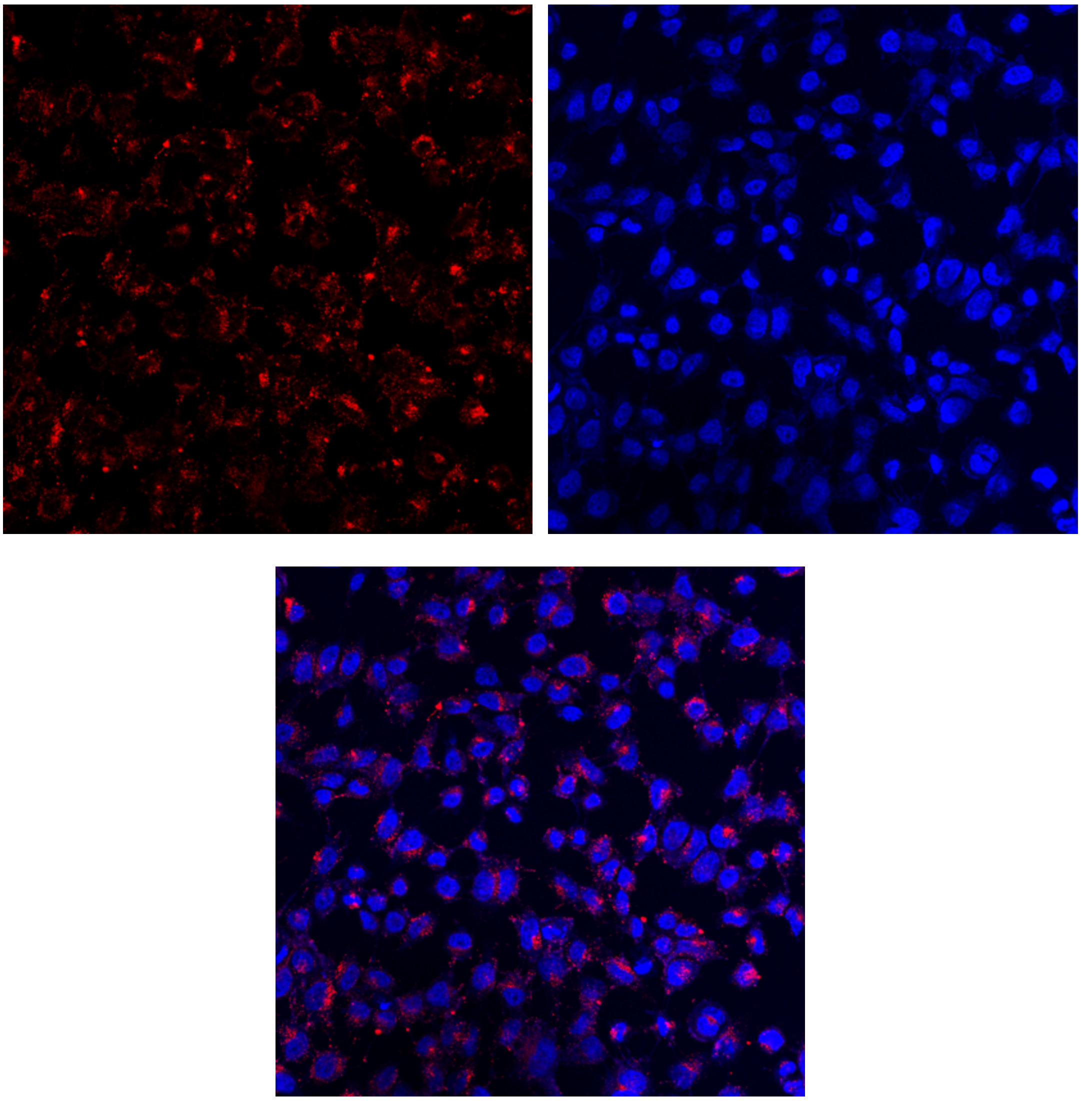

sh-SREBF2

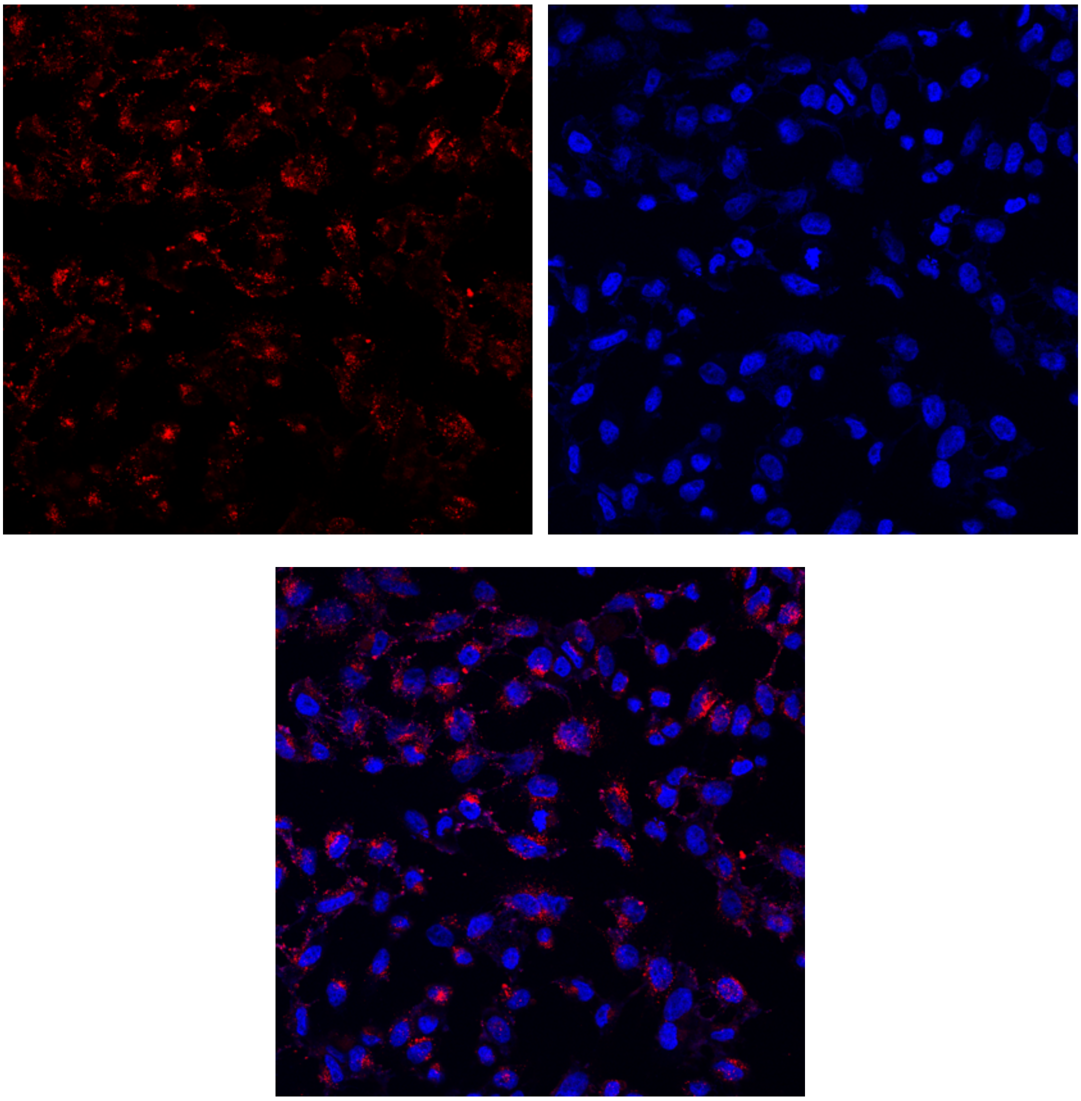

Supplement: Supplementary file 1 [file Image1.pdf]
